# A multi-machine learning framework identifies novel PANoptosis-related biomarkers and their immune landscape in ulcerative colitis: Insights from transcriptomics and experimental validation

**DOI:** 10.3389/fimmu.2026.1729942

**Published:** 2026-02-18

**Authors:** Yuan Zhao, Xiangjie Zhai, Han Wang, Sen Wang, Siliu Xu, Ni Zhu

**Affiliations:** 1 School of Stomatology and Ophthalmology, Xianning Medical College, Hubei University of Science and Technology, Xianning, China; 2 School of Biomedical Engineering and Imaging, Xianning Medical College, Hubei University of Science and Technology, Xianning, China; 3 Key Laboratory of Optoelectronic Sensing and Intelligent Control, Hubei University of Science and Technology, Xianning, China; 4 School of Pharmacy, Xianning Medical College, Hubei University of Science and Technology, Xianning, China

**Keywords:** biomarker, immune cell infiltration, machine learning, PANoptosis, ulcerative colitis

## Abstract

**Background:**

Ulcerative colitis (UC), a persistent inflammatory bowel disorder, has witnessed a gradual increase in its global incidence in recent years. This study aims to identify biomarkers linked to PANoptosis in UC, highlighting a pressing requirement to identify novel diagnostic biomarkers and therapeutic targets for improved UC management.

**Methods:**

Differentially expressed genes (DEGs) in UC were identified using R software through Gene Expression Omnibus (GEO) GSE87466 and GSE206285 datasets integration. Weighted Gene Co-expression Network Analysis (WGCNA) was employed to uncover co-expression modules. PANoptosis-related hub genes were selected using eight machine learning algorithms, followed by validation of the diagnostic markers with five machine learning algorithms in test datasets GSE38713 and GSE47908. A nomogram incorporating these six genes was subsequently constructed. Comprehensive analyses—including correlation assessment, single-cell profiling, gene set enrichment analysis (GSEA), and immune infiltration evaluation—were performed to characterize their functional relevance. Their expression profiles were further validated through DSS-induced mouse UC model.

**Results:**

Six potential biomarkers (ECSCR, IRF1, MMP1, PPARG, S100A8, S100A9) were identified, demonstrating significant upregulation or downregulation in UC. KEGG and GO enrichment analyses indicated these genes are significantly implicated in bacterial infection, immune response, and inflammation pathways. Analysis of immune cell infiltration uncovered distinct shift in immune cell composition in UC patients, correlating with the identified biomarkers. The single-cell analysis indicated that IRF1 was predominantly expressed in smooth muscle cells, while S100A8 and S100A9 showed markedly high expression in neutrophils. In the DSS-induced mouse model, all six biomarkers showed significant expression, which was consistent with their expression patterns in clinical samples.

**Conclusions:**

This study effectively discovers six PANoptosis-related biomarkers with potential diagnostic value for UC, emphasizing their role in disease progression and immune regulation, offering new biomarkers for the early diagnosis and personalized treatment of UC.

## Introduction

Ulcerative Colitis (UC) is a chronic and relapsing inflammatory bowel disease that primarily affects the colorectum, and its exact etiology has not been fully elucidated. In 2023, UC affected approximately 5 million individuals globally, with its incidence continuing to rise across regions worldwide. This upward trend in incidence—observed across diverse geographical and demographic groups—making it an important public health issue globally (1, 2). In Patients with UC are often troubled by symptoms such as diarrhea, rectal bleeding, and abdominal pain, which significantly reduce their quality of life. Additionally, the chronic nature of the disease and its recurrent episodes often lead to a substantial increase in the consumption of medical resources, imposing a significant economic burden on society and the healthcare system (2). Despite advancements in diagnostic methods and treatment strategies due to an elucidated understanding of the underlying disease mechanisms, the heterogeneous and multifactorial pathogenesis and high relapse rate remain major challenges in clinical management (1). In current clinical practice, the treatment of UC mainly relies on pharmacological and surgical interventions, including 5-aminosalicylic acid drugs, corticosteroids, immunosuppressants, and biological agents (3). Although new biological agents and targeted therapies have emerged and have improved the disease outcomes for some patients to a certain extent, a considerable proportion of patients still experience resistance to existing treatments, suboptimal efficacy, or severe adverse reactions, ultimately requiring surgical intervention (2). Furthermore, monitoring disease activity and predicting relapses mainly depend on invasive testing or nonspecific inflammatory markers, lacking highly sensitive and specific molecular biomarkers, which limits early diagnosis, precise classification, and personalized treatment of the disease. It is therefore imperative to explore new biological molecular markers to bring breakthroughs to the diagnosis and treatment of UC (1, 4).

In recent years, research on cell death has provided new perspectives for exploring the pathogenetic mechanisms of inflammatory diseases. PANoptosis, a novel inflammatory cell death process recently proposed (5, 6). Its most defining feature is the ability to simultaneously activate and coordinate the characteristics and molecular components of three classic cell death pathways—pyroptosis, apoptosis, and necroptosis—through a unified molecular platform, namely the PANoptosome complex. This process is typically triggered by strong inflammatory or infectious signals, such as specific pathogen infections or cytokine storms. It serves as a crucial defense mechanism for the body to eliminate infected or dangerous cells, while its overactivation is a core pathological driver of tissue damage in many inflammatory diseases, such as UC and sepsis (7). Its pivotal role in the pathogenesis of UC is increasingly recognized (8). The involvement of PANoptosis in UC suggests that it might play a critical role in the disease’s progression by influencing the viability of intestinal epithelial cells (IECs), which are critical for preserving epithelial barrier homeostasis (9, 10). However, research progress has been uneven. While studies have begun to delineate the molecular players of PANoptosis in UC, most evidence comes from single-dataset analyses or focuses on individual pathways (9, 10). Comprehensive biomarker discovery efforts that integrate multiple transcriptomic datasets under the PANoptosis framework—particularly those employing systematic machine-learning approaches for feature selection—remain scarce. Furthermore, the translational potential of identified candidates is often limited by a lack of validation across different analytical levels (e.g., single-cell resolution and *in vivo* models) (9). Therefore, the specific role and potential diagnostic value of PANoptosis-related molecules in UC have yet to be fully and systematically elucidated, representing a significant gap with promising clinical implications. Given the emerging evidence of PANoptosis’ role in UC, further research into this mechanism may offer valuable clinical applications and enhance our understanding of the disease.

This research investigates PANoptosis-associated molecular pathways in the pathogenesis of UC and the clinical translational potential of its related biomarkers. Unlike traditional research that approaches from a single cell death pathway, this study aims to identify PANoptosis-related molecules, attempting to break through the limitations of previous research by systematically identifying PANoptosis gene modules significantly associated with UC and exploring their role networks in disease occurrence and development. This research approach is expected to enrich the theoretical framework of UC pathogenesis and provide innovative perspectives for molecular typing and targeted interventions in inflammatory diseases (11, 12). At the technical and methodological level, this study fully integrates high-throughput data mining and multivariate statistical analysis. It first conducts differential gene screening based on publicly available expression profile data. We then used weighted gene co-expression network analysis (WGCNA) to reveal key UC-associated gene modules. On this basis, the study further employs various machine learning algorithms to select the most diagnostic PANoptosis-related biomarkers from high-dimensional features. This multi-algorithm, multi-level cross-validation strategy helps enhance the accuracy and generalizability of the screening results (13, 14). Totally, this research aims to deeply explore and validate PANoptosis-related biomarkers associated with UC through systematic bioinformatics analysis and multiple machine learning methods, providing new molecular evidence for disease diagnosis, risk stratification, and personalized treatment. Through the overall analysis of the PANoptosis signaling network and the precise identification of key molecular nodes, it is expected to promote advancements in molecular typing of UC and precision medicine, and provide references for the mechanistic research and clinical translation of related inflammatory diseases.

## Materials and methods

### Data and resources

**Figure 1:**
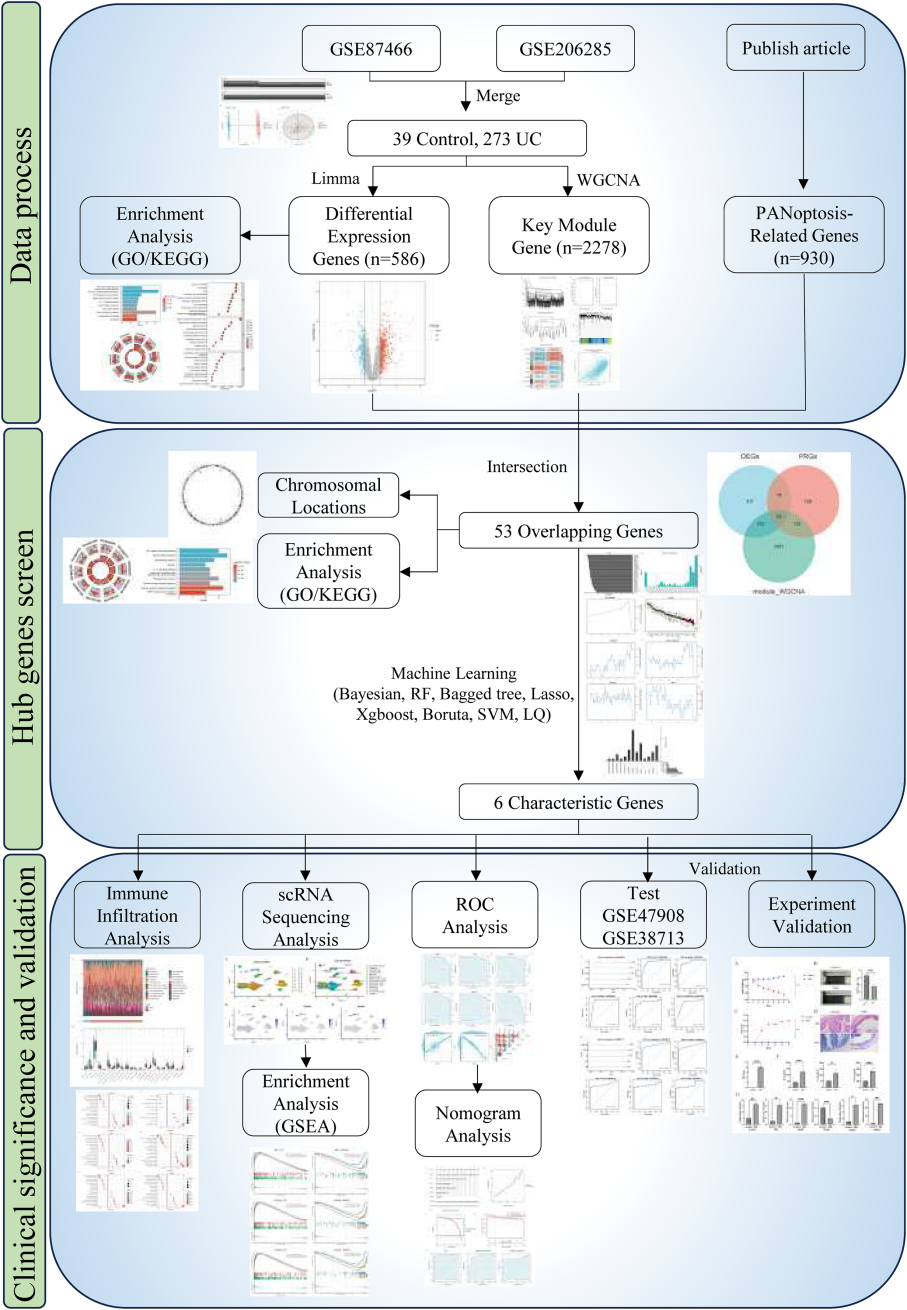
The flowchart of this study.

The overall framework of this study is shown in **Figure 1**. This study uses the R software package “GEOquery” to download the expression profiles and clinical sample grouping information of UC datasets from the Gene Expression Omnibus (GEO), including GSE87466, GSE206285, GSE38713, GSE47908, and GSE214695, with details provided in **Table 1**. The samples in the datasets are all derived from human colon tissue. GSE87466, GSE206285, GSE38713, and GSE47908 are transcriptome chip data. Probes without gene symbols were excluded. When multiple probe sets corresponded to a single gene, we selected the one with the maximum expression value for subsequent analysis. The transcriptome datasets have been batch-corrected and normalized. In this study, GSE87466 and GSE206285 are used as the test set for differential analysis of UC and for screening and modeling key genes; GSE38713 and GSE47908 serve as an independent cohort for validation of the clinical significance of key genes for UC diagnosis. The GSE214695 single-cell RNA-seq dataset enabled characterization of key gene expression distribution among distinct intestinal cell and immune cell types. In addition, this study obtained PANoptosis-related genes (PRGs) lists directly and entirely from Yang et al. (15).

**Table 1:** The basic information of GEO datasets.

Datasets	Platform	Control	Active UC
GSE87466	GPL13158	21	87
GSE206285	GPL13158	18	186
GSE38713	GPL570	13	12
GSE47908	GPL570	15	45
GSE214695	GPL18573	6	6

### Differential expression genes analysis

The “limma” package in R was applied to detect the differentially expressed genes (DEGs) in UC based on a significance cutoff of adjusted *P* < 0.05 and |log2FC| > 1. The volcano plot of the differential genes was drawn using “ggplot2”.

### Functional enrichment analysis

GO (Gene Ontology) and KEGG (Kyoto Encyclopedia of Genes and Genomes) enrichment analyses were performed to identify biologically relevant pathways and functional terms significantly associated with the candidate genes. We performed GO and KEGG enrichment analyses to elucidate the functional characteristics of differentially expressed genes. The GO analysis systematically categorized genes into biological processes (BP), cellular components (CC), and molecular functions (MF), while KEGG analysis interrogated their involvement in signaling pathways. These analyses were conducted using the “clusterProfiler” package with the “org.Hs.eg.db” annotation database, applying a significance threshold of *P*(adj) < 0.05. The resulting enrichment data were visualized through the “ggplot2,” “enrichplot,” and “GOplot” packages to facilitate biological interpretation.

### Weighted gene co-expression network analysis

WGCNA is a bioinformatics method that builds gene association networks based on high-throughput gene expression data, identifies co-expressed gene modules, and explores the association of modules with specific traits (16). This study employs the WGCNA method to analyze key gene modules closely related to UC disease. First, clustering analysis was performed on all samples to eliminate outliers. Subsequently, the co-expression network was constructed using a soft threshold of β=6, which was determined based on the scale-free topology fit (R²=0.80). The dynamic tree-cutting algorithm was used to divide genes into different modules, and similar modules were merged to obtain the final modules. By calculating the association between modules and the disease, the most significantly correlated modules were screened for further analysis.

### Machine learning-based biomarker discovery, validation, and clinical modeling

Our machine learning analysis was structured into three sequential phases to ensure a clear separation between biomarker discovery, independent validation, and clinical model translation. The overarching objective was to build a supervised learning model for distinguishing UC patients from healthy controls; therefore, the task was explicitly defined as a binary classification. All subsequent feature selection and model training were conducted within this classification framework. In the biomarker discovery phase, in order to identify a stable and informative biomarker signature from an initial candidate genes, we applied eight machine learning algorithms configured for feature importance ranking, including LASSO (using the `glmnet` package with 1000 iterations of internal 10-fold cross-validation to assess coefficient stability), alongside Support Vector Machine Recursive Feature Elimination (SVM-RFE), eXtreme Gradient Boosting (XGBoost), Learning Vector Quantization (LVQ), Bagged Trees, the Boruta algorithm (`Boruta` package), Random Forest RFE, and Bayesian RFE (`caret` package). A consensus hub genes were derived by retaining only genes selected by at least three of these eight independent methods. The subsequent validation phase evaluated the generalizable performance of this fixed gene set. Using five distinct machine learning methods (Random Forest (RF), SVM, k-Nearest Neighbors (KNN), LogitBoost, Naive Bayes (NB) via `caret`) on independent test datasets (GSE38713, GSE47908), standard 10-fold cross-validation was employed solely for hyperparameter tuning and to compute an unbiased estimate of the Area Under the Curve (AUC; using the `pROC` package), with no further feature selection. This design aimed to rigorously test the robustness and generalizability of the biomarker signature, ensuring that its high discriminative power was not due to algorithm-specific overfitting during the feature selection process but rather stemmed from its intrinsic biological informational value. In addition, to ensure a rigorous evaluation that is robust to class imbalance, we employed a comprehensive set of performance metrics beyond simple accuracy. Specifically, for each machine learning model on the validation cohorts, we calculated Balanced Accuracy, Precision, Sensitivity, Specificity, and the F1-score. Finally, for construction of a clinical prediction tool, a multivariate logistic regression model (the `rms` package) was used to build a nomogram, utilizing the expression levels of the validated genes as the sole inputs for individual risk prediction. This phased design prevents information leakage and ensures that the reported diagnostic accuracy reflects the intrinsic biological signal of the biomarkers.

### Immune infiltration analysis

Immune infiltration analysis underpins the advancement of revealing the immunopathological mechanisms of diseases, guiding patient classification and personalized treatment, and assessing disease activity and prognosis; it advances the diagnosis and understanding of diseases from the traditional level of “symptoms and biochemical indicators” to the level of “cellular and molecular mechanisms,” ultimately achieving precise intervention and treatment of immune-mediated diseases. This study employs the CIBERSORT method (https://cibersort.stanford.edu/) to assess the infiltration abundance across 22 distinct immune cell populations, excluding cells with an abundance of zero. The “ggplot2” package is used to create stacked bar charts, group comparison plots, and lollipop charts related to immune infiltration.

### Single-cell transcriptome analysis

Single-cell transcriptomics is mainly used to accurately parse cellular heterogeneity, discover new cell types, reconstruct cellular developmental trajectories, and analyze the “social networks” between cells. Single-cell data analysis has brought biological research from the vague era of “tissue average levels” into the high-definition era of the “cellular universe.” This study utilizes the “Seurat,” “scDblFinder,” “harmony,” and “SingleR” packages to analyze the GSE214695 single-cell dataset, which includes 3 UC and 3 control datasets. The single-cell data undergoes various processing steps including quality control, double cell handling, cell cycle processing, cell clustering and annotation, integration, and dimensionality reduction, followed by differential analysis of PANoposis-associated genes using the single-cell transcriptomic data and plotting.

### Gene set enrichment analysis

Gene Set Enrichment Analysis (GSEA) interprets gene expression data from a systems perspective, revealing the underlying biological pathways and functional changes. Unlike enrichment analyses based on differential analysis, such as GO and KEGG, GSEA focuses more on detecting the overall changes in a specific pathway or biological process across the entire expression profile. GSEA was conducted using the “clusterProfiler” package with the “org.Hs.eg.db” annotation database. We defined significantly enriched pathways as those exhibiting a normalized enrichment score (NES) > 2 and a false discovery rate (FDR) q-value < 0.25. Visualization was conducted using the “enrichplot” package.

### Establishment of DSS-induce colitis in mice model

Male C57BL/6 mice (8 weeks old, 20–22 g, n=12) were housed under specific pathogen-free (SPF) conditions. All animal procedures were reviewed and approved by the Animal Ethics Committee of Hubei University of Science and Technology (No. 2025-03-104). Mice were randomly divided into two groups: the model group (n=6) received 3.5% (w/v) dextran sulfate sodium (DSS) dissolved in drinking water ad libitum for 7 days, while the control group (n=6) received sterile distilled water under the same conditions. During the administration period, disease symptoms associated with UC in mice were monitored, and disease severity was assessed using the disease activity index (DAI) (17). On day 7, all mice were euthanized by cervical dislocation. Whole blood and colon tissue samples were collected for further analysis.

### Colonic tissue pathological morphological observation

Pathological changes in mouse colonic epithelial tissue: After tissue fixation, embedding, paraffin sectioning (thickness 4 μm), baking in an oven, and dewaxing with xylene and gradient ethanol. Hematoxylin-eosin (HE) staining was performed, with hematoxylin staining for 3 min, followed by rinsing with tap water, differentiating solution for 20 s, rinsing with tap water, eosin staining for 1 min, and rinsing with running water, finally using a blue solution for 30 s until the tissue turned blue, followed by rinsing with running tap water for a few seconds. Dehydrated, cleared, and mounted with neutral gum. Observed under a light microscope, examining tissue morphological structure, with colonic tissue damage index (TDI) referring to the literature (17). Alcian Blue-periodic acid-Schiff (AB-PAS) staining was employed to assess goblet cells morphology and distribution in mouse colonic tissue: tissue fixation, embedding, sectioning, dewaxing, dehydrating, clearing, and mounting, all performed similarly to HE staining. AB-PAS staining was performed according to the manufacturer’s protocol. Briefly, sections were stained with Alcian Blue for 10 min, followed by three 3-min washes. After oxidation in periodic acid solution (5 min) and tap water rinsing, slides were treated with Schiff Reagent for 10 min and then rinsed under running tap water for 10 min. Counterstaining was performed with hematoxylin for 1 min, followed by three rinses and differentiation in acid alcohol for 30 s, before final rinsing. Finally, dehydrated, cleared, and mounted with neutral gum, observed under a light microscope (18).

### Inflammatory cytokine detection

Levels of IL-6, IL-1β, and TNF-α in serum and colon homogenates were measured by enzyme-linked immunosorbent assay (ELISA) using specified kits per manufacturer’s instructions (Shanghai Enzyme-linked Biotechnology Co., Ltd., ml063159, ml098416, ml002095), with absorbance read at 450 nm and concentrations calculated against standard curves.

### RNA extraction and quantitative real-time PCR

Total RNA was isolated from colon tissues using the FastPure Cell/Tissue Total RNA Isolation Kit V2 (Vinozan, China). Subsequently, 1 μg of total RNA was reverse-transcribed into cDNA with HiScript III RT SuperMix for qPCR (+gDNA wiper). Quantitative PCR was performed using Taq Pro Universal SYBR qPCR Master Mix on a real-time PCR system, with cDNA as template to determine the expression levels of the six target genes. All primer sequences used are listed in **Table 2**, and the reaction setup and cycling conditions followed the manufacturer’s protocols. Gene expression levels were quantified using the 2^(^–ΔΔCt^) method.

**Table 2:** RT-qPCR primer information.

Gene name	Gene sequence accession number	Forward primer (5’ to 3’)	Reverse primer (5’ to 3’)
ECSCR	NM_001033141.2	AACACTGACACCCCTTCTTGCC	TGCGAGGTCTGAGTTGTCGT
IRF1	NM_001159393.1	CGACACACATCGATGGCAAG	CAGAAAGCCAGCAAAAGACTCC
MMP1	NM_008607.2	GGTCCAGGCGATGAAGACCCC	GGGTGCAGGCGCCAGAAGAA
PPARG	NM_011146.4	TGCTGTTATGGGTGAAACTCTG	CTGTGTCAACCATGGTAATTTCTT
S100A8	NM_013650.2	TGAACTGGAGAAGGCCTTGAG	AGAGGGCATGGTGATTTCCT
S100A9	NM_009114.3	AGGAAGGAAGGACACCCTGA	GCTCAGCTGATTGTCCTGGT
GAPDH	NM_001411842.1	GTGTTCCTACCCCCAATGTG	GTCATTGAGAGCAATGCCAG

### Statistical analysis

All statistical computations were performed with R (v4.3.0) or GraphPad Prism 9.0 software. Experiment data were presented as Mean ± Standard Deviation. The student t-test was employed for statistical treatment. Data with *P* < 0.05 were considered to be significant.

## Results

### Screening of DEGs in UC

**Figure 2:**
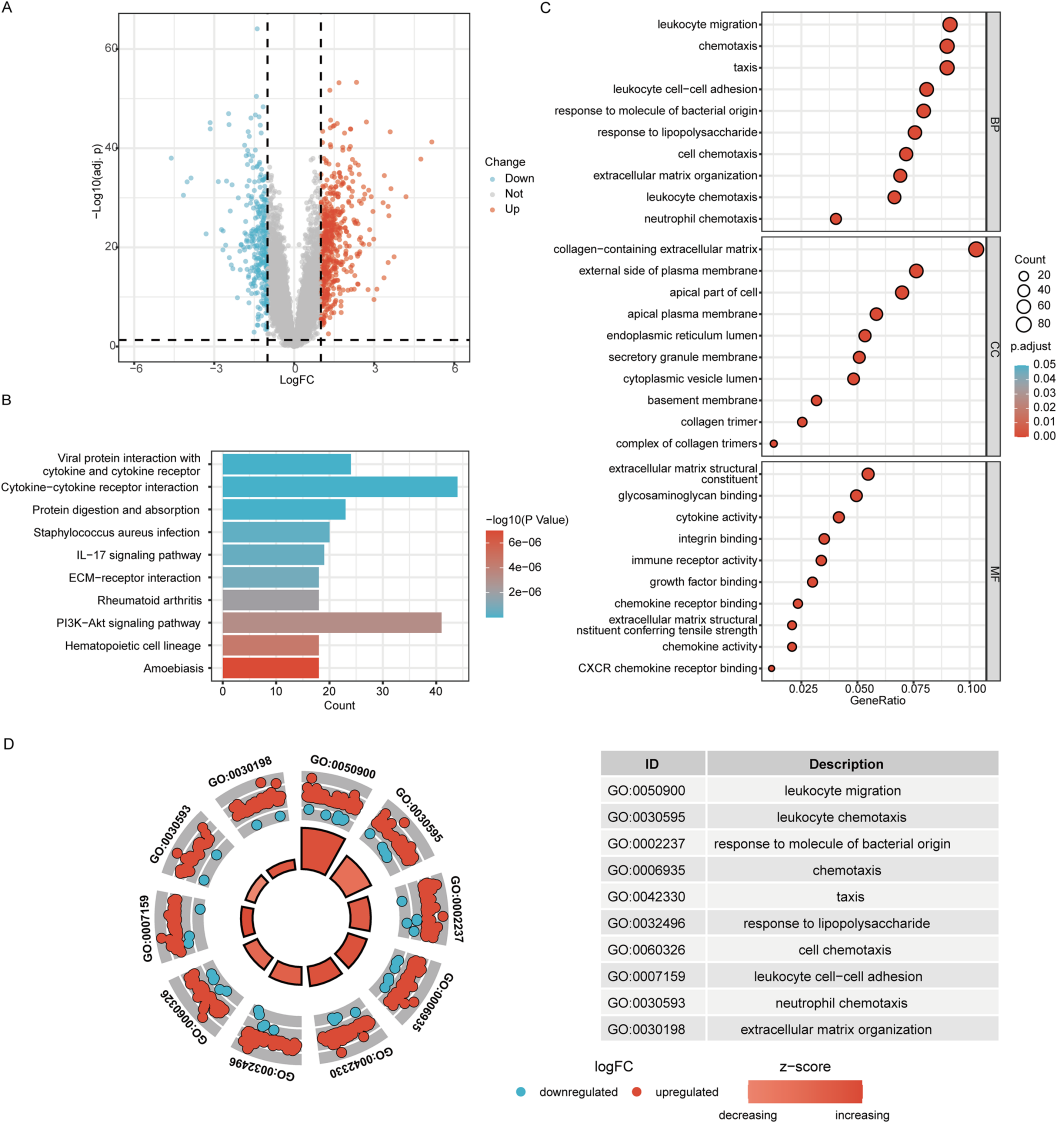
DEGs of UC and their functional enrichment analysis was performed. **(A)** The volcano plot showed 858 DEGs between the UC and Control groups in the GSE206285 and GSE87466 combined dataset. B-D. The enrichment analysis of DEGs was performed by KEGG **(B)**, GO **(C)** and GO combined with log FC **(D)**.

To obtain DEGs in UC, the datasets GSE206285 and GSE87466 were first merged, batch effects were removed, and normalization was performed. Ultimately, expression profile data from 39 control samples and 273 UC samples were included for subsequent analysis (**Supplementary Figure S1**). Differential expression analysis of the merged transcriptome data was conducted with the limma package, comparing UC samples against controls. This analysis identified 858 DEGs, comprising 537 upregulated and 321 downregulated genes. **Figure 2A** presents the volcano plot visualizing the DEGs. Functional profiling of the DEGs through KEGG and GO enrichment analyses is depicted in **Figures 2B-D**. KEGG enrichment analysis identified the top 10 significantly enriched pathways among the 858 DEGs, including cytokine-cytokine receptor interaction, PI3K-Akt signaling, and IL-17 signaling pathway (**Figure 2B**; **Supplementary Table S1**). The results of the GO enrichment analysis (**Figure 2C**; **Supplementary Table S2**) and the combined GO with logFC (Circular visualization of GO enrichment with logFC value, **Figure 2D**) indicated that significant involvement of these DEGs in multiple biological processes, including leukocyte migration, chemotaxis, leukocyte cell-cell adhesion, response to molecules of bacterial origin, response to lipopolysaccharide, etc. GO analysis mapped the DEGs to specific cellular components including collagen−containing extracellular matrix, external side of plasma membrane, apical part of cell, apical plasma membrane, endoplasmic reticulum lumen, etc., while molecular function categorization featured terms such as extracellular matrix structural constituent, glycosaminoglycan binding, cytokine activity, integrin binding, and immune receptor activity.

### Gene co-expression modules in UC

**Figure 3:**
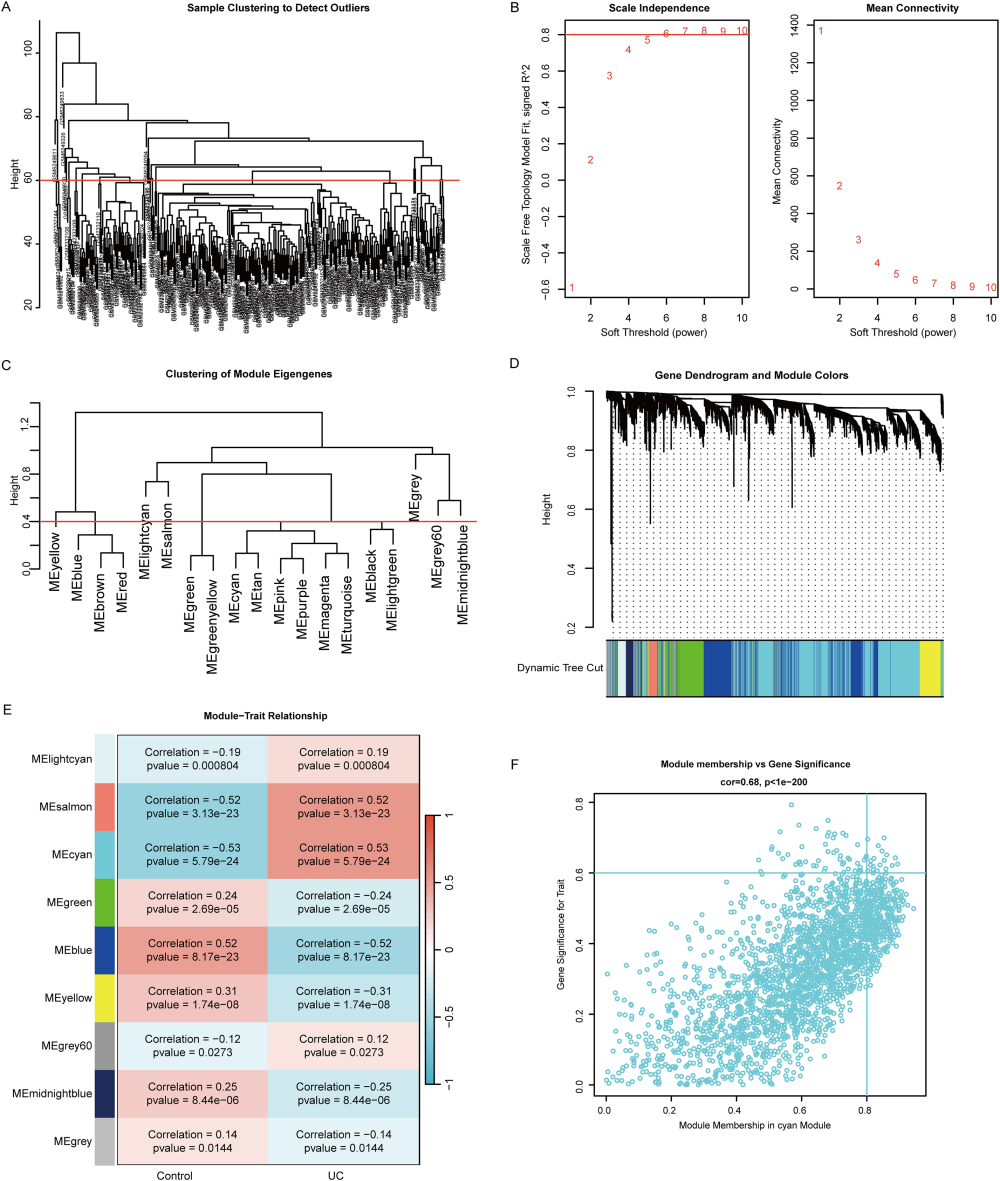
Construction of weighted co-expression network analysis in UC. **(A)** A cluster sample tree of 312 samples in UC. **(B)** Scale-free topology analysis for the optimal soft thresholding power (β). **(C)** Hierarchical clustering dendrogram of eigengenes. **(D)** Gene dendrograms using the average linkage criterion. **(E)** Module gene heatmap. **(F)** A scatterplot of gene significance for UC vs. module membership in the cyan module.

Using WGCNA analysis to identify co-expressed modules in UC. First, data processing of samples was performed to calculate the variance of each gene, checking for missing entries, entries with weights below the threshold, and genes with zero variance, removing poor genes and samples. Subsequently, the “average correlation degree” method was used to cluster the samples, with the cutting line set at 60. The sample clustering tree (cluster the samples tree) is shown in **Figure 3A**. Next, we constructed biologically significant scale-free networks with a scale independence of > 0.80 and a soft threshold power β=6 (**Figure 3B**). Through hierarchical clustering analysis and dynamic TreeCut, 9 gene co-expression modules were identified (**Figures 3C, D**). The results of module-trait correlation analysis indicated that the cyan module was significantly positively correlated with UC (correlation=0.53, *P* < 0.05), therefore we chose this module for further in-depth study (**Figure 3E**). The cyan module contains 2278 genes, and the scatter plot in **Figure 3F** shows a strong correlation between these genes and UC (correlation=0.68, *P* < 0.05).

### Selecting PANoptosis-related hub genes in UC

**Figure 4:**
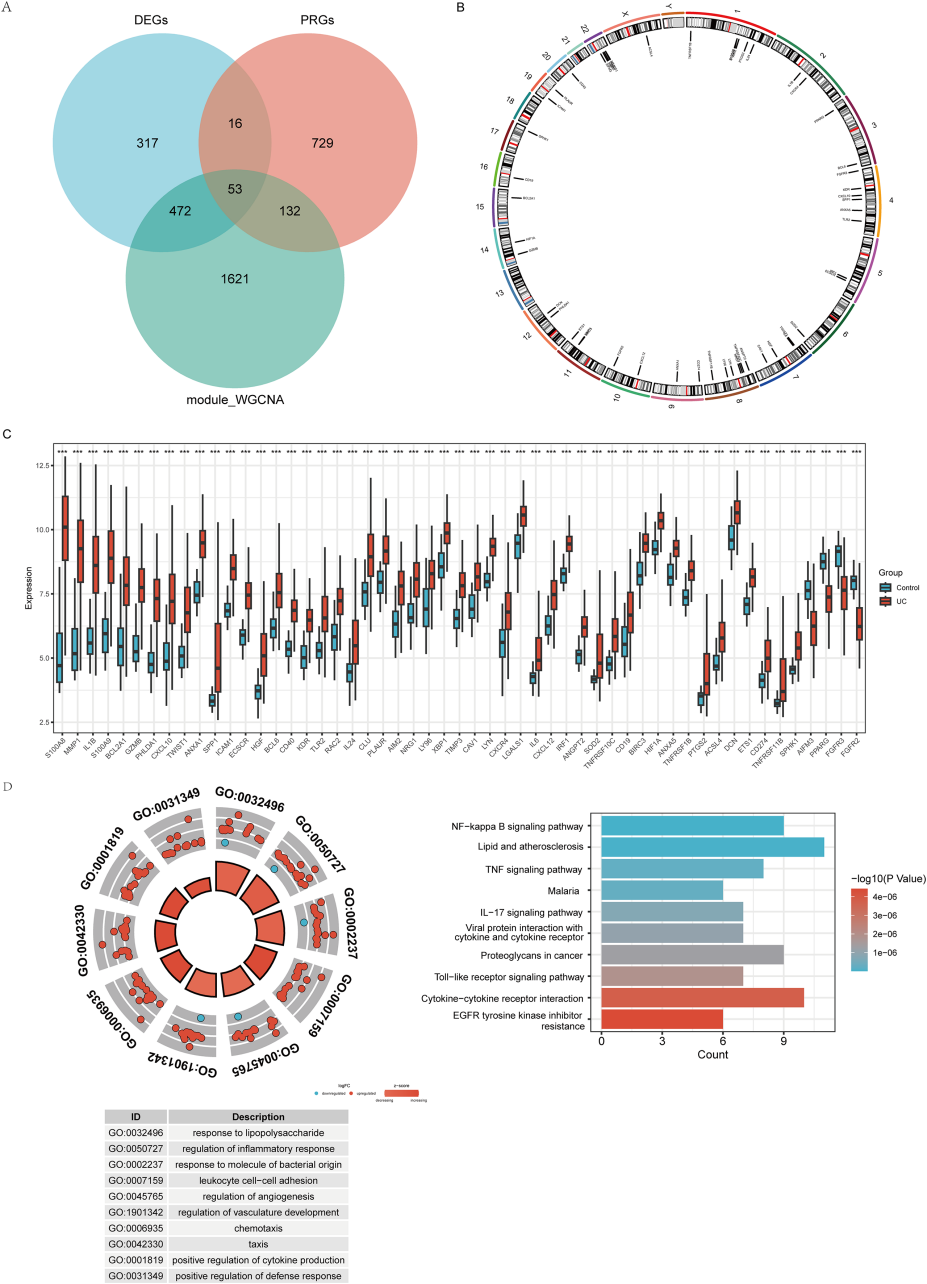
Selecting 53 key genes as PANoptosis-related hub genes in UC. **(A)** Venn diagram of DEGs, cyan module genes and PANoptosis related genes. **(B)** Chromosome circle plot of 53 PRGs-UC. **(C)** The boxplot of 53 PRGs-UC expression levels. **(D)** GO combined logFC and KEGG enrichment of 53 PRGs-UC.

To screen for key genes related to PANoptosis in UC, we used Venn diagram to examine the overlap of DEGs, cyan module genes, and PANoptosis-related genes (PRGs) in the UC test dataset. The results showed that there are 53 shared genes among the three, designated as “PANoptosis-related hub genes in UC” (PRGs-UC, **Figure 4A**). **Figure 4B** shows the positions of these 53 genes on human chromosomes. Compared to the control group, 49 of the 53 genes were upregulated in UC, while the AIFM3, PPARG, FGFR3, and FGFR2 genes were downregulated in UC (**Figure 4C**). GO combined LogFC and KEGG analysis of these 53 genes showed that their biological functions are mainly related to the response to lipopolysaccharide, regulation of inflammatory response, response to molecules of bacterial origin, leukocyte cell-cell adhesion, and regulation of angiogenesis, among others related to bacterial infection and immune response processes. KEGG results showed that these genes are highly associated with inflammation-related pathways (NF-κB, TNF, IL-17, and TLR pathways, **Figure 4D**; **Supplementary Table S3-4**).

### Machine learning screening and validation of diagnostic markers

**Figure 5:**
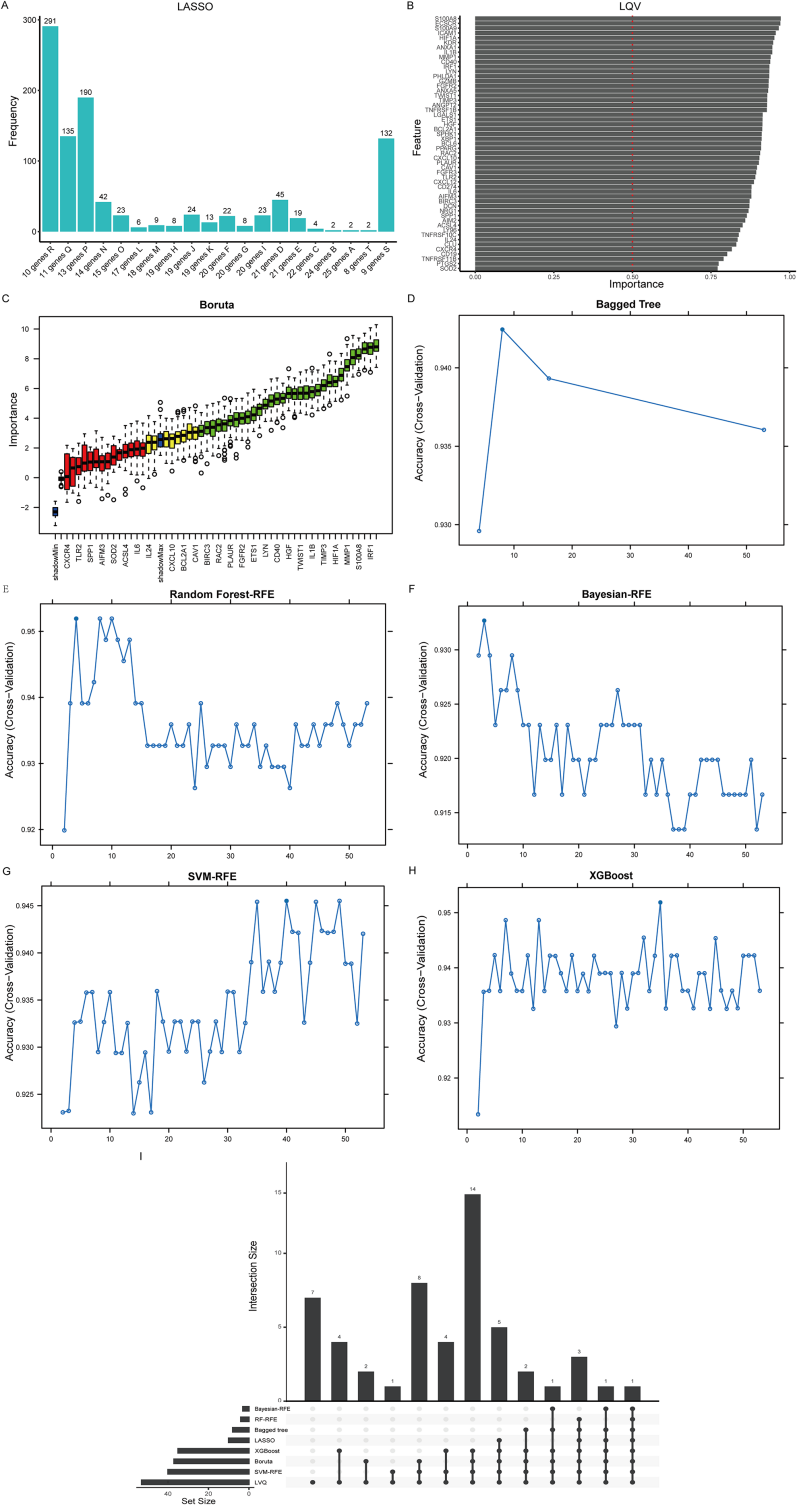
Detection of novel biomarkers using machine-learning algorithms within a binary classification framework (UC vs. Control). **(A)** Screening biomarkers based on the LASSO regression algorithm. **(B)** Screening biomarkers based on the LQV regression algorithm. **(C)** Screening biomarkers based on the Boruta algorithm. **(D)** Screening biomarkers based on the Bagged Tree algorithm. **(E)** Screening biomarkers based on the Random Forest-RFE algorithm. **(F)** Screening biomarkers based on the Bayesian-RFE algorithm. **(G)** Screening biomarkers based on the SVM-RFE algorithm. **(H)** Screening biomarkers based on the XGBoost algorithm. **(I)** UpSet visualization for screening of diagnostic biomarkers in PRGs-UC.

**Figure 6:**
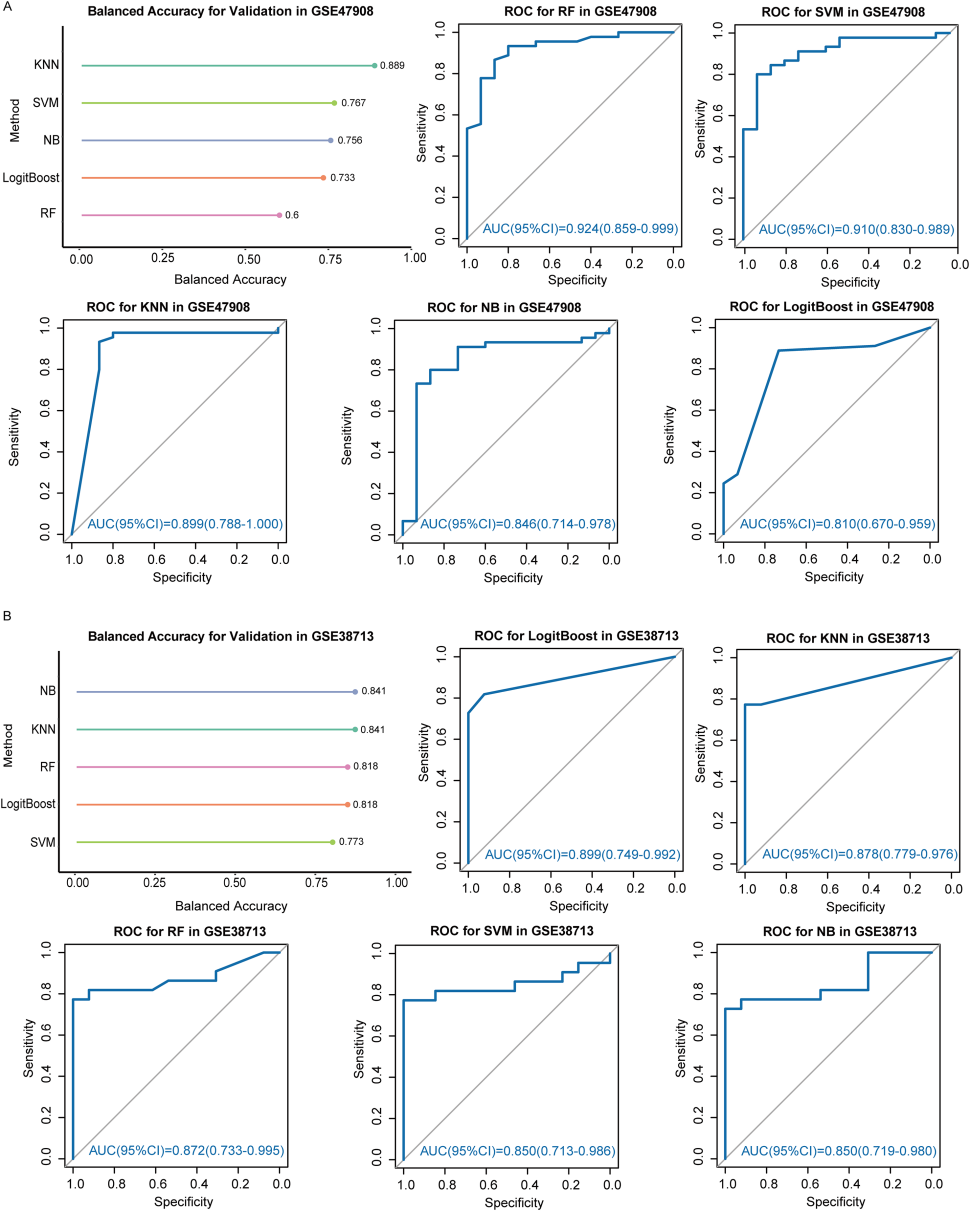
Validation of novel biomarkers using machine-learning algorithms. **(A)** Performance evaluation of five machine-learning algorithms for UC vs. Control classification on the GSE47908 dataset. **(B)** Performance evaluation of five machine-learning algorithms for UC vs. Control classification on the GSE38713 dataset.

To identify candidate diagnostic biomarkers for UC from the 53 PRGs, we applied eight machine learning algorithms with different selection principles (LASSO, LVQ, Boruta, Bagged Trees, Random Forest-RFE, Bayesian-RFE, SVM-RFE, and XGBoost, **Figures 5A-H**). The consensus analysis, requiring selection by at least six algorithms, yielded a stable set of six PANoptosis-related genes: ECSCR, IRF1, MMP1, PPARG, S100A8, and S100A9 (**Figure 5I**). We then rigorously evaluated the diagnostic robustness of this six-gene signature. To directly address potential concerns about class imbalance—a known challenge in our cohort—we moved beyond conventional accuracy. Instead, we prioritized Balanced Accuracy as our primary metric for validation, as it equally weights the model’s ability to identify both UC and Control. Using five additional classifiers (RF, SVM, Knn, NB, LogitBoost) on two independent validation sets (GSE47908 and GSE38713, **Figure 6**), the signature performed consistently well. The key finding is that high discrimination was maintained under this stringent assessment. In GSE47908, the top model achieved a balanced accuracy of 0.889 (AUC: 0.899, 95% CI: 0.799-1.000), with a corresponding F1-score of 0.857. Similarly strong results were observed in GSE38713, the top model achieved a balanced accuracy of 0.841 (AUC: 0.850, 95% CI: 0.719-0.980), with a corresponding F1-score of 0.788. The complete set of performance characteristics, including precision and F1-score for all models, is provided in **Supplementary Table S5**). All models were developed and evaluated within the binary classification framework established in our study.

### Construction of Nomogram diagnostic model and validation

**Figure 7:**
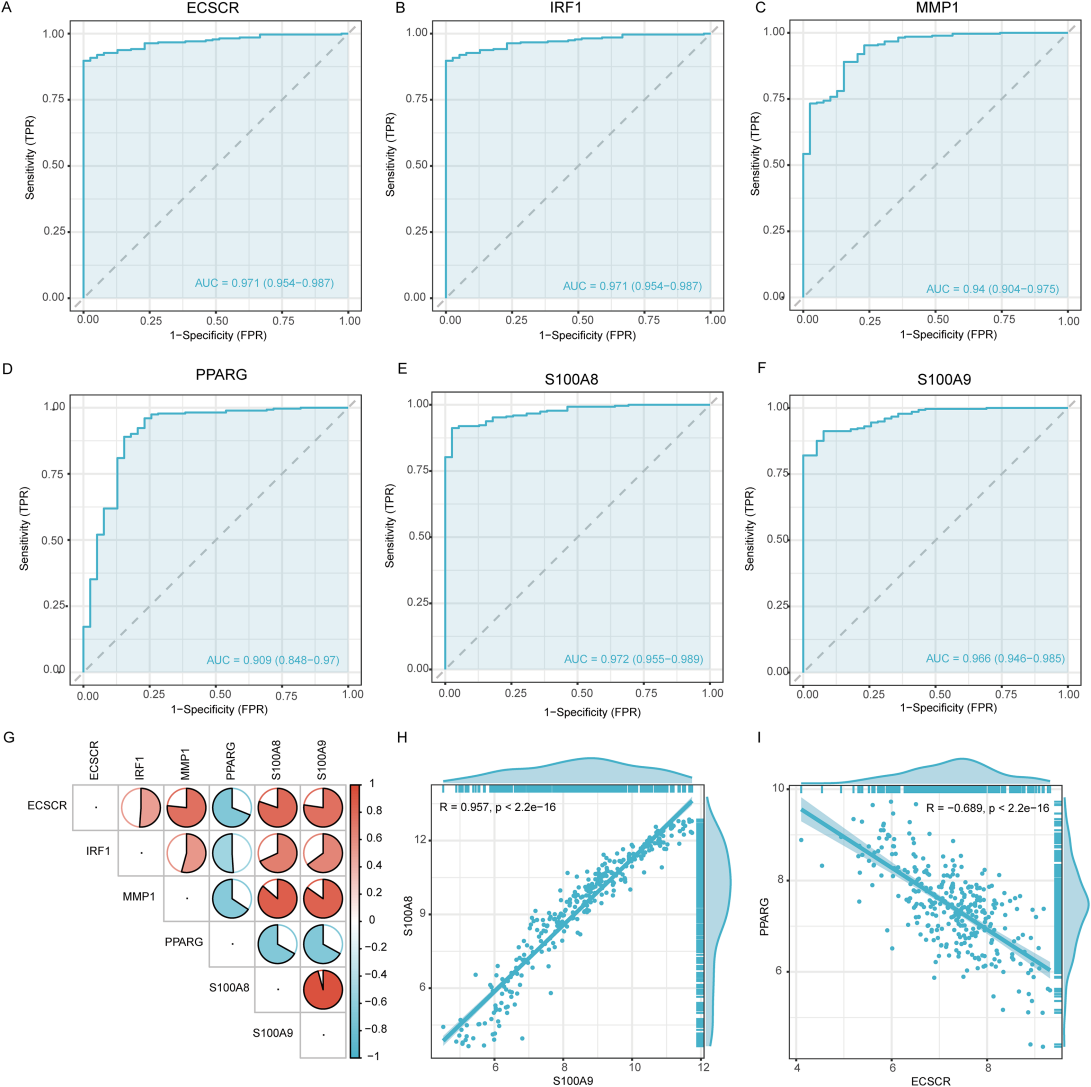
Validation of novel biomarker genes expression in UC. **(A-F)** ROC curves for ECSCR, IRF1, MMP1, PPARG, S100A8, and S100A9 in train dataset. **(G)** Correlation analysis of six biomarker genes. **(H)** The positive correlation between S100A8 and S100A9. **(I)** The negative correlation between PPARG and ECSCR.

**Figure 8:**
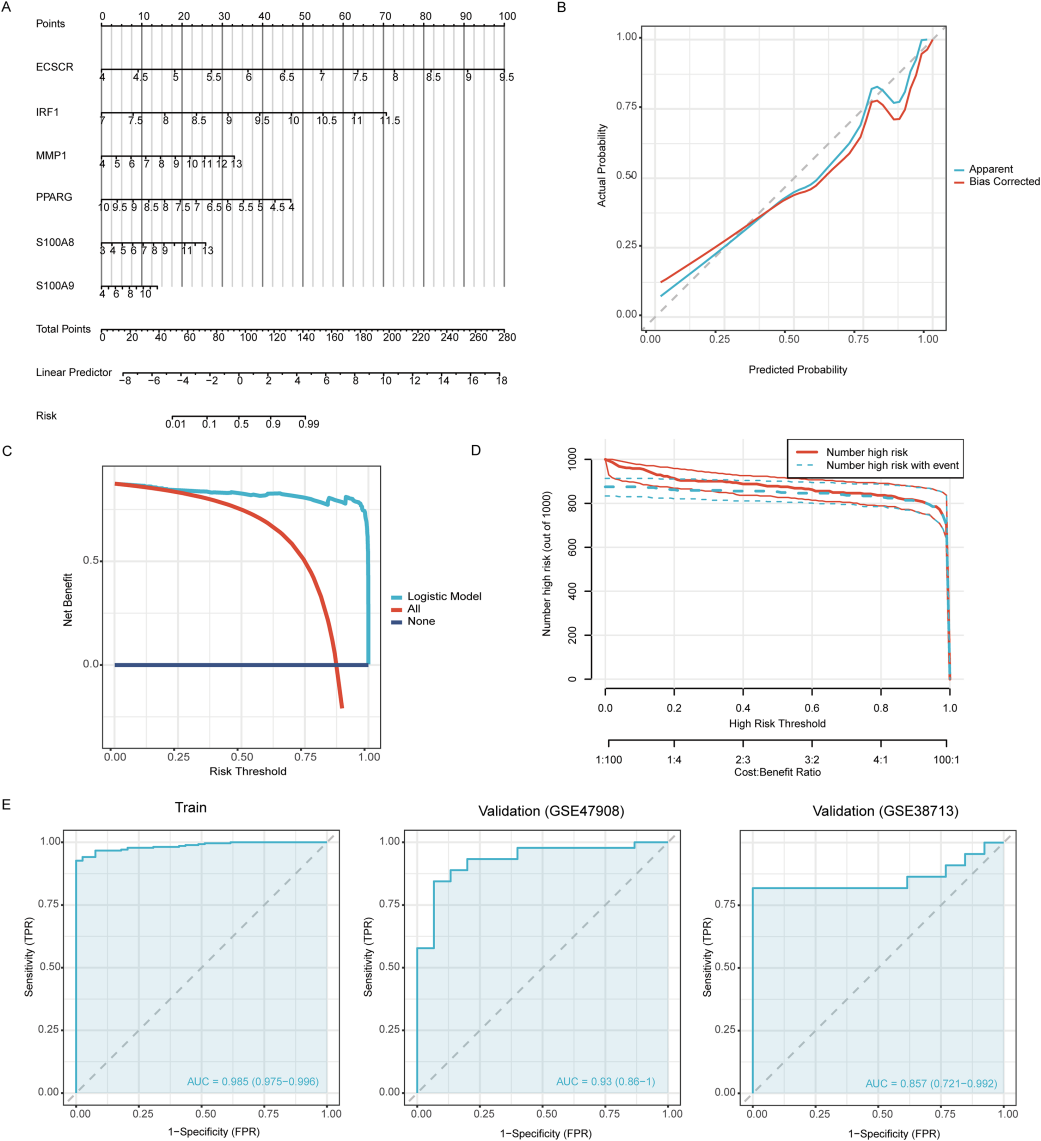
Nomogram model construction to evaluate UC risk. **(A)** Nomogram model of 6 hub genes in UC. **(B)** Calibration curve. **(C)** DCA analysis. **(D)** Clinical impact curve. **(E)** ROC analysis of model in train, GSE47908 and GSE38713.

The diagnostic efficacy of these six genes was validated in the test set through ROC curve analysis (**Figure 7**); all genes exhibited AUC values greater than 0.900. These results indicate that the 6 genes selected have strong diagnostic value for UC. **Figure 7G** analyzes the expression correlations among these six genes within the UC patient cohort. A high degree of co-expression was observed, with S100A8 and S100A9 showing a strong positive correlation (R²=0.957, **Figure 7H**). Notably, the expression level of PPARG was strongly negatively correlated with that of ECSCR (R²=-0.689, **Figure 7I**). A nomogram UC diagnostic model was constructed using these 6 biomarkers, as shown in **Figure 8A**. **Figures 8B-D** validates this model, and the results indicate that the model fits well. Further validation of the model’s diagnostic value is shown in **Figure 8E**, with the results indicating that in the training set, AUC = 0.985, and the diagnostic value is also good in the validation sets GSE47908 (AUC = 0.93) and GSE38713 (AUC = 0.857), suggesting the reliability of this UC diagnostic model.

### The landscape of immune cell infiltration

**Figure 9:**
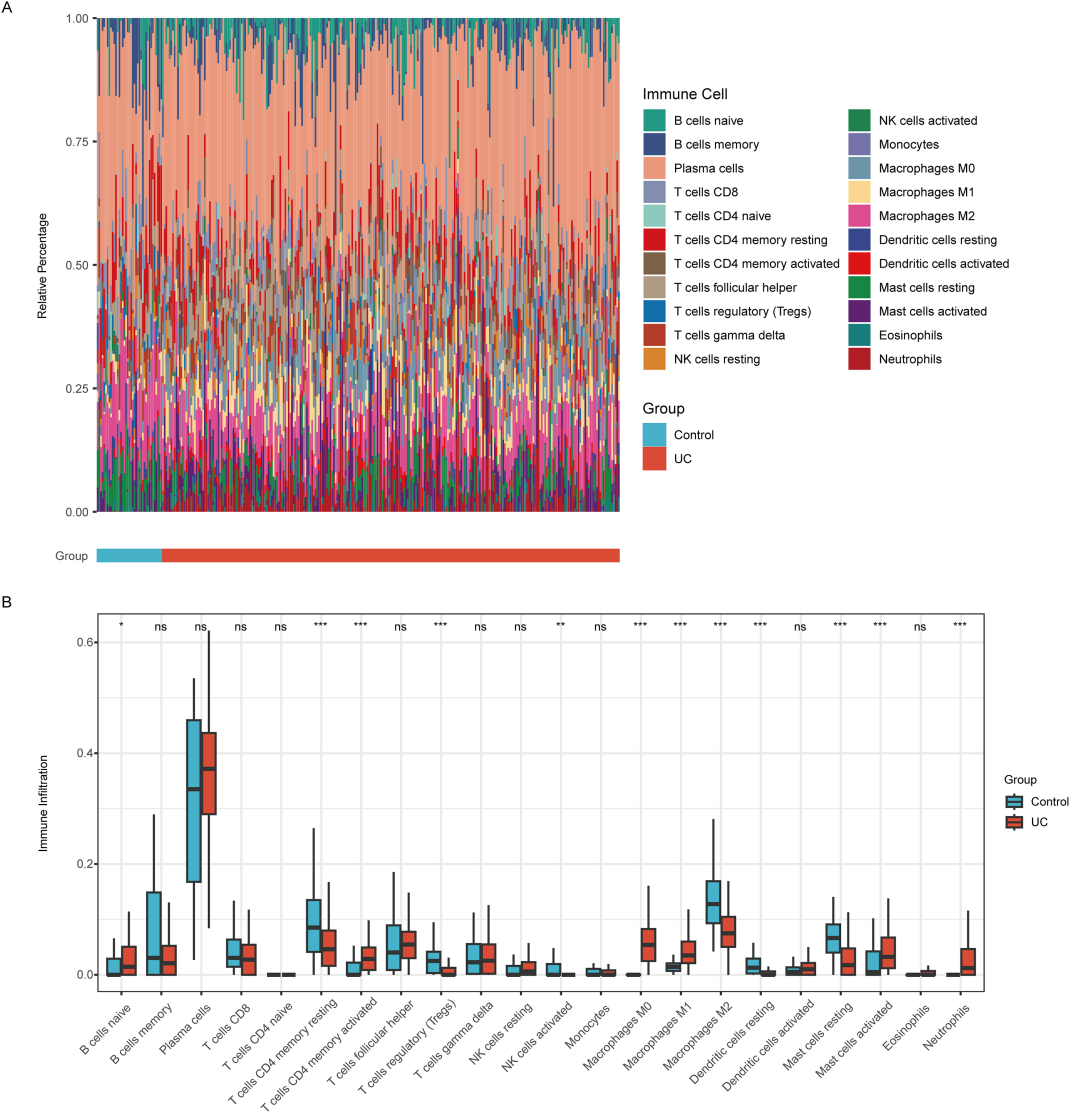
Immune cell infiltration analysis. **(A)** The proportion of immune cells in the UC and Control groups. **(B)** The CIBERSORT-based boxplot indicates differences in the levels of 22 immune cells between the Control and UC groups.

**Figure 10:**
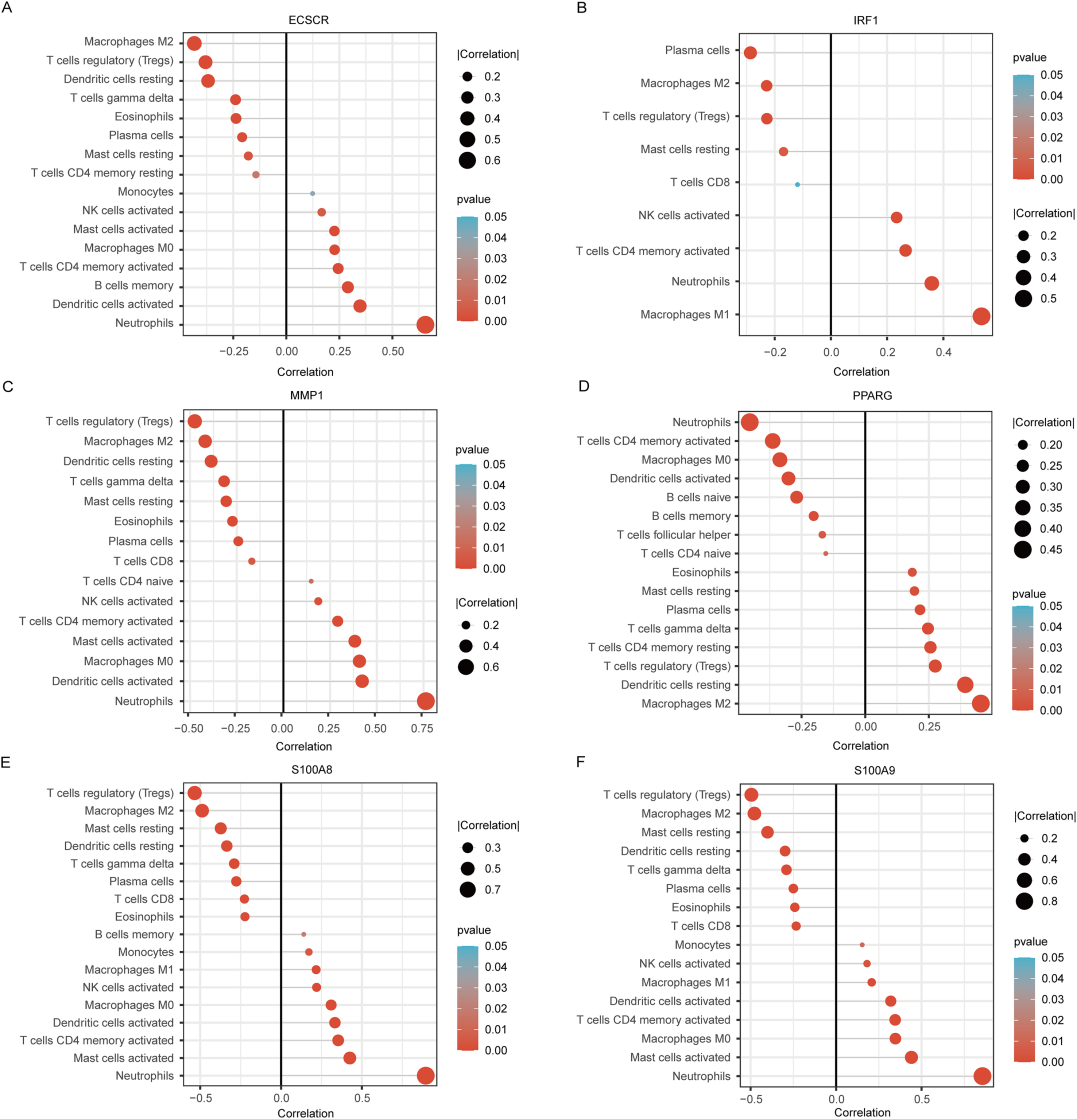
Correlation between 6 biomarkers and infiltrating immune cells. **(A)** Correlation between ECSCR and infiltrating immune cells. **(B)** Correlation between IRF1 and infiltrating immune cells. **(C)** Correlation between MMP1 and infiltrating immune cells. **(D)** Correlation between PPARG and infiltrating immune cells. **(E)** Correlation between S100A8 and infiltrating immune cells. **(F)** Correlation between S100A9 and infiltrating immune cells.

Immune cell infiltration patterns were analyzed using CIBERSORT, revealing distinct differences in the immune landscape between UC and Control groups. These findings are presented in **Figure 9**. Our results indicate that the UC group exhibits significant infiltration of various immune cells, including naïve B cells, resting CD4 memory T cells, activated CD4 memory T cells, regulatory T cells (Tregs), activated NK cells, M0 macrophages, M1 macrophages, M2 macrophages, resting dendritic cells, resting mast cells, activated mast cells, and neutrophils. Correlational analysis between the six candidate genes and immune cell infiltration revealed strong associations with multiple immune cell types (**Figure 10**).

### Single-cell profiling and GSEA analysis of hub genes

**Figure 11:**
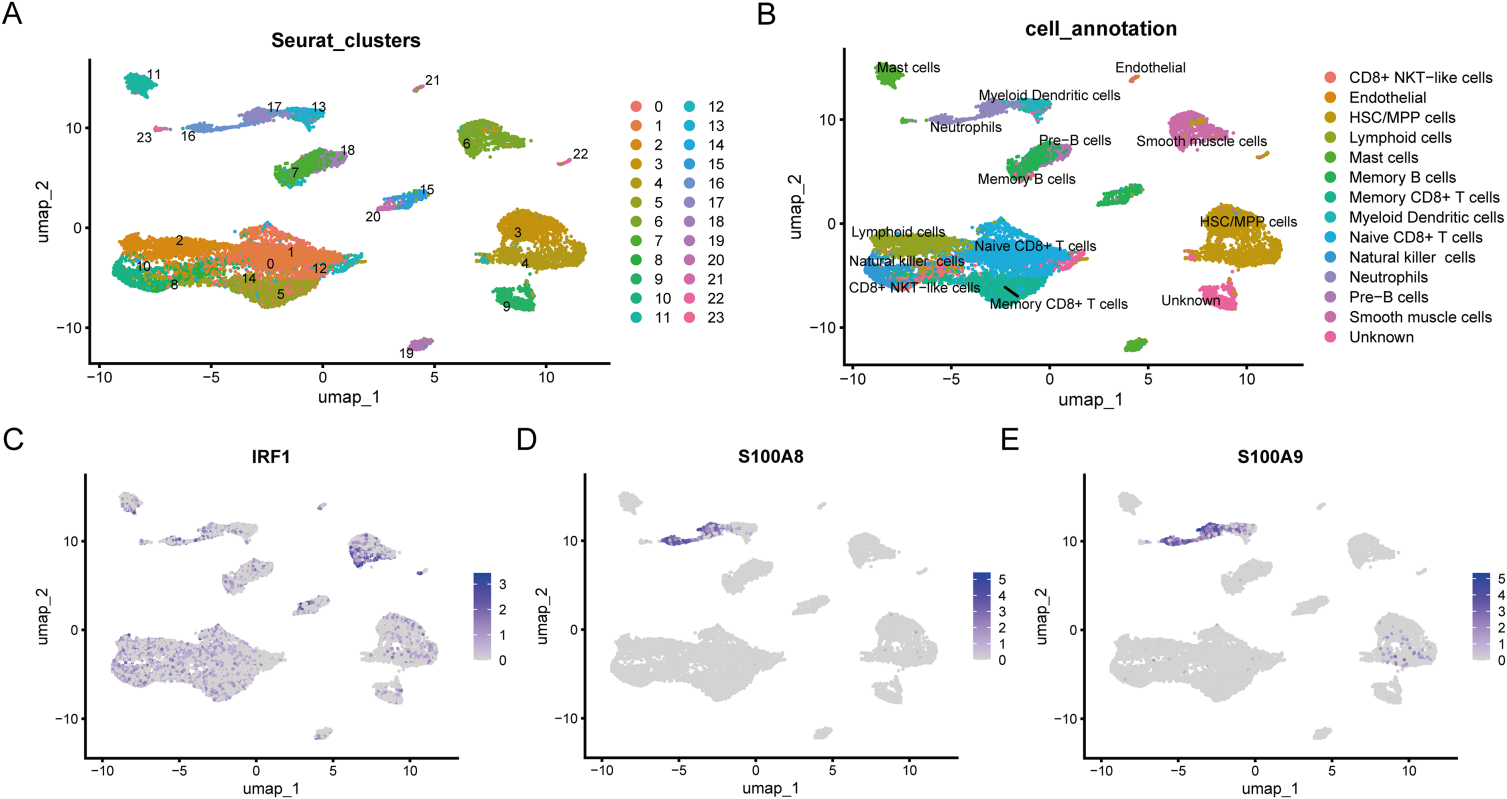
Expression profiles of hub genes in single cells. **(A, B)**. The cells from 6 samples were clustered into 24 subsets. Colors indicate the cell types. **(C-E)**. Scatter plots of the expression of the 3 hub genes, including IRF1 **(C)**, S100A8 **(D)**, and S100A9 **(E)**.

**Figure 12:**
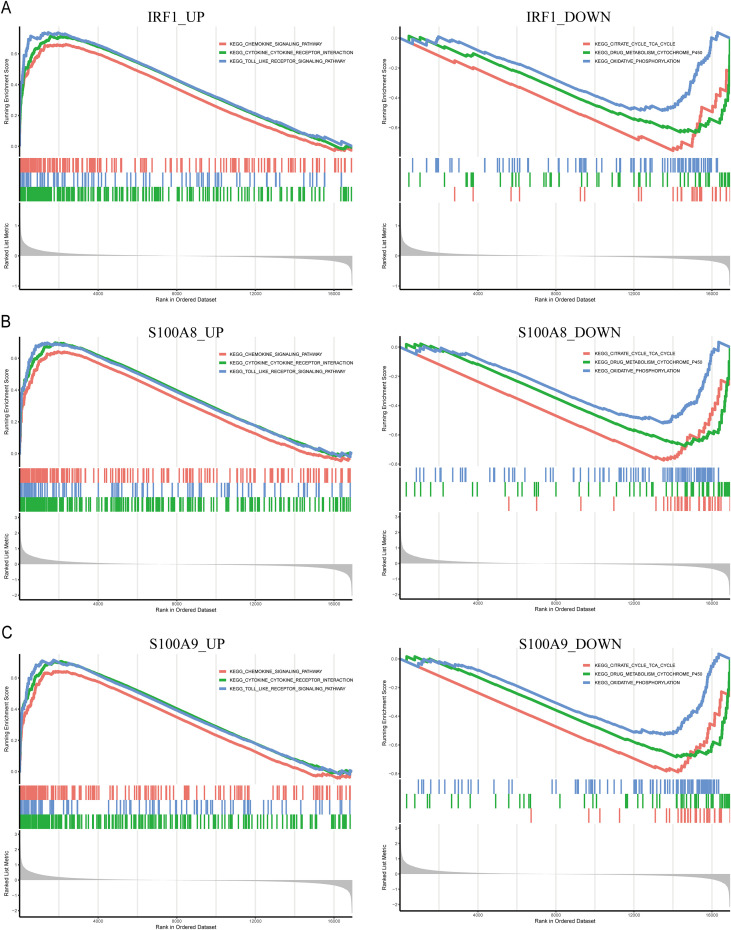
GSEA analysis of three hub genes. **(A)** GSEA enrichment results of IRF1. **(B)** GSEA enrichment results of S100A8. **(C)** GSEA enrichment results of S100A9.

Single-cell transcriptomic analysis was performed on three control and three UC colon samples from dataset GSE214695. Unsupervised clustering identified 24 distinct cell populations, which were annotated using established marker genes according to previous literature (**Figures 11A, B**). Our analysis characterized 13 distinct cell populations, comprising CD8+ NKT-like cells, Endothelial, HSC/MPP cells, Lymphoid cells, Mast cells, Memory B cells, Memory CD8+ T cells, Myeloid Dendritic cells, Naïve CD8+ T cells, Natural killer cells, Neutrophils, Pre-B cells, and Smooth muscle cells. Among the 6 identified biomarker genes, only IRF1, S100A8, and S100A9 were found to be significantly expressed in specific cell types, such as: IRF1 being highly expressed in Smooth muscle cells, and S100A8 and S100A9 primarily being significantly expressed in Neutrophils (**Figures 11C-E**). Our single-cell resolution analysis reveals the precise cellular localization of hub genes within distinct colonic cell populations during colitis pathogenesis. To decipher their potential functional impacts of IRF1, S100A8, and S100A9 in UC, we employed a gene-centric GSEA strategy (**Figure 12**). The results indicated that the KEGG pathways positively correlated with IRF1, S100A8, and S100A9 in UC mainly include chemokine, cytokine-cytokine receptor interaction, and TLR pathways, while the KEGG pathways negatively correlated with the three are: Citrate cycle TCA cycle, drug metabolism cytochrome P450, and Oxidative phosphorylation.

### Hub genes expression in DSS-induce UC

**Figure 13:**
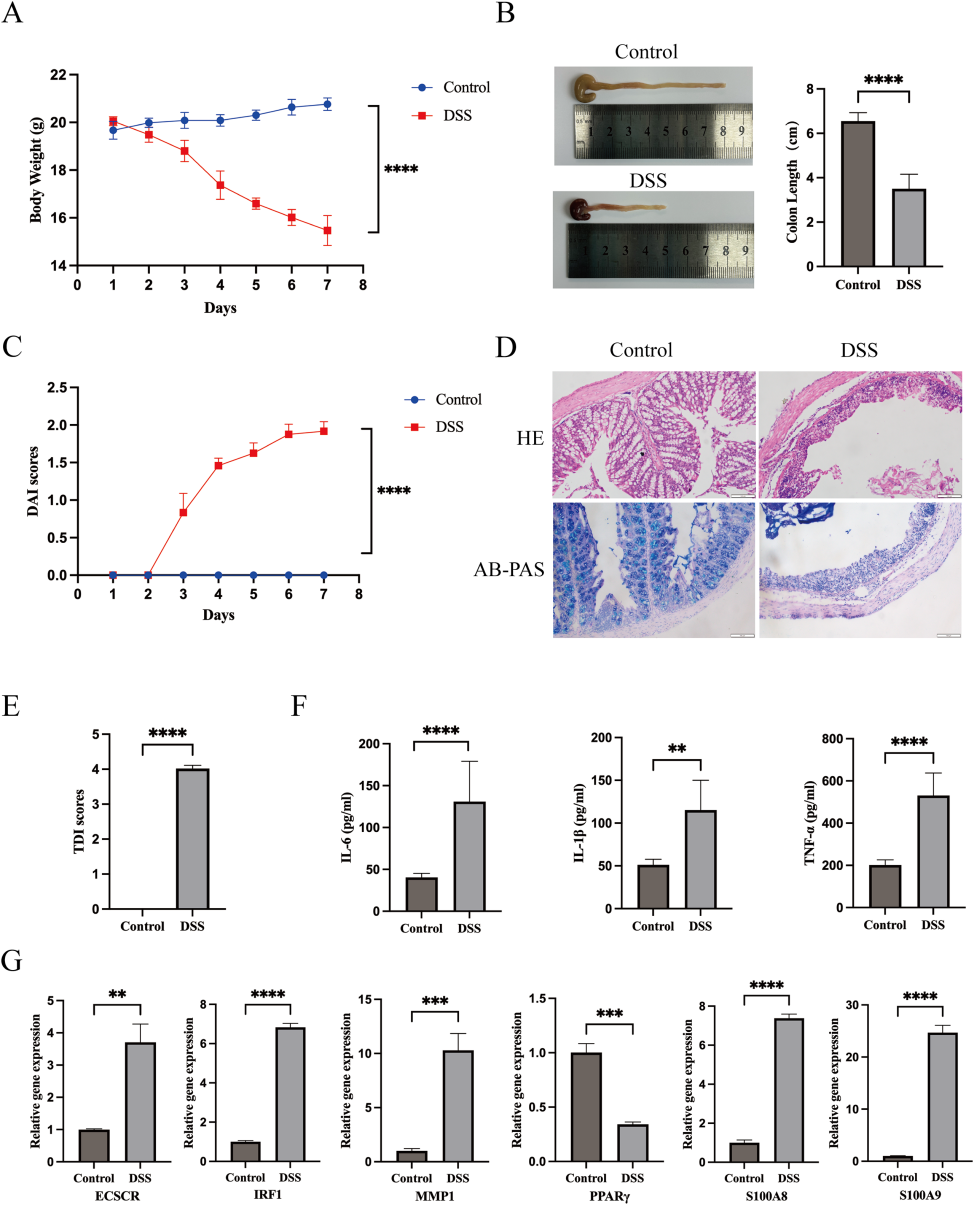
Hub genes expression in DSS-induced ulcerative colitis in mice. **(A)** The body weight changes in DSS-induced ulcerative colitis mice. **(B)** The colon length changes. **(C)** the disease activity index (DAI). **(D)** The HE and AB-PAS staining of colon. **(E)** The tissue damage index (TDI) scores. **(F)** The inflammatory cytokines (IL-6, IL-1β and TNF-α) changes in serum of DSS-induced ulcerative colitis mice. **(G)** The mRNA expression levels of 6 hub genes in DSS-induced ulcerative colitis mice. In comparison with the control group, a statistically significant result was observed at **P < 0.01, ***P < 0.001, and ****P < 0.0001.

To experimentally validate hub gene expression in UC, we established a well-established DSS-induced colitis model in mice. As shown in **Figure 13**, after 7 days of DSS treatment, there was a significant decrease in mouse body weight (**Figure 13A**), a significant reduction in colon length (**Figure 13B**), and a significant increase in the DAI (**Figure 13C**). HE and AB-PAS staining results (**Figure 13D**) showed that the mucosal structure, goblet cells, and crypts of the colon tissue in the control group mice were clear and intact, with the structure of the colonic epithelial cells being distinctly visible, glands arranged neatly, and no inflammatory infiltration. Histological examination revealed marked inflammatory cell infiltration densely distributed throughout the mucosal and submucosal layers, accompanied by a reduction in goblet cells and the disappearance of crypt structures in DSS-treated mice. The TDI significantly increased (**Figure 13E**). The levels of IL-1β, IL-6, and TNF-α in the serum of DSS-treated mice were significantly elevated (**Figure 13F**), indicating that DSS successfully induced the establishment of the UC model. Experimental quantification by qPCR confirmed transcriptional changes of the six hub genes in colonic tissues (**Figure 13G**). These results were concordant with the GEO data, showing PPARG downregulation and upregulation of the remaining five genes. Specifically, IRF1, S100A8, and S100A9 showed substantial induction, with expression values in DSS-administered mice measuring more than sixfold higher than in the control group.

## Discussion

UC is a chronic inflammatory bowel disorder that causes severe symptoms in patients. These include diarrhea, abdominal pain, and other digestive issues. The causes of this disease are complex and involve many factors, including genetic background, environmental influences, and imbalances in the immune system of the intestines. Despite the availability of several treatment options, issues such as limited effectiveness and significant side effects remain, prompting researchers to explore new biomarkers to improve early detection and treatment of the disease. This study aims to elucidate the role of biomarkers associated with PANoptosis related to UC by integrating machine learning techniques to select genes related to UC and evaluate their potential effectiveness as biomarkers. In this research, we successfully identified six PANoptosis-related biomarkers, including ECSCR, IRF1, MMP1, PPARG, S100A8, and S100A9, which demonstrated significant diagnostic potential for UC. Our integrated machine learning framework enabled the systematic identification of these biomarker candidates, underscoring the value of computational approaches in biomedical research. Our findings suggest that these biomarkers not only correlate with UC but also provide insights into the underlying pathophysiological mechanisms. The robust performance of these biomarkers in ROC analyses indicates their potential clinical utility, paving the way for improved diagnostic strategies in UC management.

Regarding the expression of PANoptosis-related genes and their molecular mechanisms in UC, this study identified a significant hub gene community related to PANoptosis through screening with R software and combining the WGCNA method. The concept of PANoptosis, integrating pyroptosis, apoptosis, and necroptosis, has recently garnered significant attention in inflammatory bowel disease (IBD) research. Previous studies have established that various forms of programmed cell death in intestinal epithelial cells are abnormally active in UC, contributing to epithelial barrier dysfunction and immune imbalance (9). Earlier bioinformatics analyses have begun to explore this field. For instance, Wang et al. conducted a comprehensive analysis of a PANoptosis-related gene signature in UC, identifying key components like ZBP1, AIM2, and CASP1 through differential expression and PPI network analysis (9). Another study by Wu et al. applied machine learning (LASSO and SVM-RFE) to integrated periodontitis and UC datasets, identifying BAG3, LYN, and APOE as shared core genes linked to PANoptosis, highlighting its potential role in inflammatory comorbidities (19). These studies, along with others focusing on single cell death pathways (e.g., ferroptosis or necroptosis), often relied on single or limited datasets, conventional statistical methods, or lacked integration of multi-omics validation. The intestinal mucosa of UC patients exhibits highly active PANoptosis, an integrated cell death process linked to prominent infiltration of pro-inflammatory immune cells (20, 21). PANoptosis’ significant activation in the intestinal mucosa of UC patients exacerbates epithelial cell death, leading to impaired barrier function and immune imbalance (9, 20). The 53 key genes identified in this study are closely related to inflammatory response and immune cell infiltration, further supporting the pivotal role of PANoptosis in regulating the immune microenvironment and promoting disease progression (21, 22). Compared to existing high-throughput analyses, this study systematically reveals the synergistic effects within the PANoptosis molecular network and its driving effects on inflammatory cascade responses through the WGCNA network modular approach, expanding the understanding of the integration of “synergistic death” signals at the molecular mechanism level. Notably, some studies focus on the downstream effects of a single PANoptosis pathway, whereas this study emphasizes the interactions among multiple genes within modules and their integrated effects in processes such as immune cell recruitment and cytokine signal amplification, thereby providing novel insights into the complex molecular interplay underlying UC pathogenesis (19, 23). Unlike previous studies that were based solely on small-scale chips or single-factor analysis, this research captures potential co-disruption mechanisms of hub genes in PANoptosis, thereby providing a more precise biological basis for elucidating the complex inflammation-cell death interactions in UC. Recent advances continue to underscore the relevance of our findings. A 2024 review by Sun et al. elaborates on the mechanistic roles of PANoptosis in disease, supporting our focus on this integrated pathway (11). Furthermore, a 2025 bioinformatics study by Lu et al. also employed machine learning to analyze PANoptosis and autophagy in UC, reinforcing the growing trend of using advanced computational methods to dissect cell death pathways in IBD (23). Another 2025 study by Wan et al. combined machine learning with immune infiltration analysis to characterize PANoptosis-related biomarkers, paralleling our approach but identifying a different gene set, which highlights the heterogeneity of UC and the value of diverse analytical strategies (22). Our study contributes to this evolving landscape by providing a validated, multi-faceted signature and a translatable risk score model. In summary, this study deepens the understanding of the core pivotal mechanisms of PANoptosis-related genes in the course of UC through bioinformatics methods.

During the biomarker screening phase, the integration of eight machine learning algorithms for feature selection of PANoptosis-related hub genes significantly improved the accuracy and robustness of candidate biomarker screening. Previous studies mostly relied on single models or traditional statistical methods, such as LASSO, SVM, RF, which struggled to fully capture the nonlinear features and complex interactions within high-dimensional transcriptome data (24, 25). Our study employed an advanced, three-phase machine learning framework designed to maximize robustness, generalizability, and clinical translatability, which constitutes a significant methodological advancement. In the biomarker discovery phase, we applied a stringent consensus approach, leveraging eight distinct feature selection algorithms (LASSO, SVM-RFE, XGBoost, LVQ, Bagged Trees, Boruta, Random Forest RFE, and Bayesian RFE) to identify a stable core gene signature. This multi-algorithmic consensus strategy critically mitigates the bias inherent in relying on any single feature selection method. Subsequently, in the independent validation phase, the diagnostic performance of this fixed gene set was rigorously evaluated using five distinct classifiers (Random Forest, SVM, k-Nearest Neighbors, LogitBoost, Naive Bayes) on separate cohorts, employing a comprehensive suite of metrics (AUC, Balanced Accuracy, F1-score, etc.) to ensure robust evaluation. Finally, this validated signature was translated into a clinical prediction tool via a multivariate logistic regression nomogram. This phased, multi-model integration—encompassing extensive feature screening, independent performance validation, and clinical model construction—provides a more systematic and rigorous evaluation framework compared to studies utilizing limited methodological combinations (e.g., LASSO, SVM, RF alone). It effectively minimizes overfitting, enhances the reliability of the identified biomarkers, and ensures their diagnostic utility is derived from intrinsic biological signal rather than algorithmic artifact. Unlike previous strategies that relied solely on co-expression analysis or single algorithm screening, the approach in this study can automatically adapt to different data structures, avoid overfitting, and enhance generalization ability, particularly maintaining excellent performance even with limited sample sizes. Additionally, some researchers report that diagnostic models related to PANoptosis-related genes suffer from insufficient generalization and limited specificity. However, the cross-validation strategy of multiple algorithms in this study effectively overcomes these limitations (21, 26). Therefore, the diversified application of machine learning methods not only enriches the technical pathways for biomarker screening but also provides methodological innovations for the precise identification of molecular features in complex diseases.

For the external validation of biomarkers, this study applied five machine learning methods to assess the diagnostic capabilities of the six selected biomarkers in an independent validation set, showing stable predictive performance across different datasets and sample backgrounds. Unlike previous studies that only performed internal validation within the training set, this research further examined the model’s generalizability and clinical applicability through an independent validation set (21, 27). Literature reports indicate that the expression heterogeneity of PANoptosis-related markers is high across different populations and sample types, leading to the failure of some models on external data (23). By alternately using different machine learning algorithms to construct and test models, this study found that core biomarkers maintained high sensitivity and specificity across various diagnostic scenarios, indicating that the selected genes possess strong cross-platform stability and adaptability. Additionally, the clinical predictive value was further quantified by combining decision curves and ROC analysis, contrasting sharply with previous approaches that validated using a single indicator or algorithm, significantly enhancing the credibility of the results (22). The above results suggest that PANoptosis-related biomarkers can achieve stable and reproducible diagnostic discrimination under different validation conditions, promising to provide a more objective molecular tool for the early identification and classification of UC. This stage of strategy has strengthened the clinical translational foundation of the candidate biomarkers.

This study systematically integrates six biomarkers with patient clinical features based on a nomogram model for the clinical modeling and correlation analysis of biomarkers, quantifying the independent contributions of each indicator in disease diagnosis prediction. In recent years, the nomogram combined with multivariable molecular markers has gradually been applied in various inflammatory diseases and tumors to assist with diagnosis and prognosis stratification (28). In the field of UC, some existing models are mainly based on traditional laboratory or imaging indicators, lacking visual prediction tools for the integration of multiple molecular markers at the molecular level (29, 30). This study innovatively incorporates PANoptosis-related molecular characteristics into the nomogram, significantly improving the model’s predictive accuracy and interpretability. Unlike previous studies that focused solely on the predictive ability of individual molecular markers, this research emphasizes the advantages of integrating molecular and clinical information in modeling (31–33). The literature shows that the nomogram, as a multi-factor visual modeling tool, can enhance the precision of individualized risk assessment by integrating multiple sources of information, including molecular, clinical, and laboratory data (32). The nomogram model in this study demonstrated high calibration and discrimination in both internal and external testing, indicating the practical usability and guiding value of core biomarkers in clinical diagnostic processes. Compared to previous single-gene or single-indicator models, the nomogram that integrates multiple PANoptosis markers can more comprehensively reflect the molecular heterogeneity of UC patients, facilitating clinicians in formulating individualized treatment strategies based on different molecular risk phenotypes (34, 35). Therefore, the nomogram model based on PANoptosis biomarkers provides a new avenue and tool for precise risk prediction in UC.

The analysis of immune cell infiltration in UC patients revealed significant alterations in the composition of immune cells, which are closely associated with the identified biomarkers. Specifically, the results indicated that the UC group exhibited a marked increase in various immune cell types, including activated T cells, regulatory T cells, and macrophages, compared to the control group. This shift in immune cell dynamics suggests a robust immune response that may contribute to the pathogenesis of UC. The correlation between immune cell types and the identified biomarkers underscores their potential role in the disease’s immunoregulation. For instance, the presence of specific immune cells such as CD4+ T cells and macrophages may influence the inflammatory milieu, thereby affecting disease progression and severity. The findings from this study not only enhance our understanding of the immune landscape in UC but also highlight the potential of these biomarkers as diagnostic and therapeutic targets. By elucidating the relationship between immune cell infiltration and disease markers, this research paves the way for developing personalized treatment strategies aimed at modulating the immune response in UC patients, ultimately improving clinical outcomes and quality of life. The integration of immune profiling with biomarker identification could lead to more effective monitoring and management of UC, addressing the unmet needs in current therapeutic approaches (1).

While single-cell analysis successfully mapped key biomarkers like IRF1 and S100A8/A9 to specific cellular contexts, the non-detection of ECSCR and MMP1 primarily stems from a fundamental methodological divergence. Bulk RNA-seq robustly identifies diagnostic signatures based on consistent, aggregate expression across tissue—an approach validated by the independent, computational rediscovery of ECSCR as a UC biomarker (36). In contrast, single-cell sequencing, while powerful for resolving heterogeneity, is inherently limited in detecting low-abundance or sporadically expressed transcripts. Therefore, the absence of a signal at single-cell resolution does not negate its biological or diagnostic relevance established at the bulk tissue level. Ultimately, the machine learning model selected a synergistic gene panel, not a collection of individual markers. The panel’s strong and reproducible discriminative power (AUC > 0.80 in independent cohorts) confirms that its diagnostic validity is derived from the concerted contribution of all six genes.

In the animal experimental validation phase, this study conducted empirical testing of the expression of six key genes in UC model animals, revealing that all exhibited significant regulatory changes in UC pathogenesis. ECSCR is a cell-surface protein predominantly localized to endothelial cells, with physiological functions involving cell migration, signal transduction, and metabolic regulation. ECSCR shows promise as a UC biomarker, with markedly elevated serum levels that maintain a distinct correlation with neutrophil infiltration, and associated with the clinical classification of UC and the assessment of drug efficacy, indicating its potential important value in for the clinical translation of UC biomarkers (36). Our results also confirm this finding. IRF1 acts as a pivotal transducer that converts upstream interferon signaling into the execution of downstream PANoptosis. This dual role positions it in the pathogenesis of infectious diseases, autoimmune diseases (such as systemic lupus erythematosus), and inflammatory diseases (such as UC). It serves as both a crucial host defense molecule and a potential driver of excessive inflammation and tissue damage. In the process of the occurrence and development of UC, the upregulation of IRF1 expression is causally confers increased susceptibility to the disease; the high expression of IRF1 may exacerbate the pathological progression of UC by promoting immune and inflammatory processes (37). Among the PANoptosis-related genes, IRF1 is also considered one of the molecular markers for UC (9), consistent with this study. The dysregulation of MMP1, a key enzyme in extracellular matrix turnover, may be associated with UC progression. Increased MMP1 expression may indicate active tissue remodeling and inflammatory processes in the intestinal mucosa. In UC, MMP1 is a high-performance, inflammation-related diagnostic biomarker. It not only contributes to improved diagnostic accuracy, but its potential role in disease pathogenesis—such as driving tissue degradation and inflammation—also makes it an important molecule for understanding UC pathological mechanisms and developing personalized treatment strategies (38). Existing research indicates that the expression and function of PPARG undergo significant changes in UC patients, and its impaired activity may lead to the persistence and worsening of intestinal inflammation. Activating PPARG can alleviate UC symptoms and restore its function (39). The S100 family proteins S100A8 and S100A9 function as key regulators of inflammation and innate immunity. Their expression is largely confined to myeloid cells such as neutrophils and monocytes, where they act as central mediators of immune responses. S100A8 and S100A9 act as inflammatory mediators in UC, affecting host immune responses and exacerbating disease progression by altering gut microbial composition and metabolism (40, 41). S100A8 and S100A9 are key molecules in UC that serve a dual role as both “diagnostic tools” and “pathogenic factors.” Clinically, they serve as non-invasive and reliable intestinal inflammation biomarkers, significantly optimizing the diagnosis, disease activity assessment, and therapeutic management of UC. In basic research, the essential role of S100a8/a9 is to act as a “bridge” connecting upstream neutrophil inflammation with downstream endothelial cell PANoptosis. By systematically disrupting mitochondrial function and homeostasis, it ultimately releases the decisive signal (mtDNA) that activates the ZBP1-PANoptosis pathway, leading to devastating disruption of the vascular endothelial barrier in inflammatory diseases such as sepsis. Therefore, targeting S100a8/a9 or its downstream pathways is considered a key strategy for intervening in this destructive process (42). This study’s animal experiments confirm the dynamic expression changes of selected biomarkers in the UC model, which is highly consistent with the aforementioned molecular mechanisms report. Additionally, the regulatory patterns of some genes show a positive correlation with disease severity, further supporting their feasibility as targets for disease progression and treatment. Unlike previous validations that were limited to *in vitro* or artificially overexpressed/knockout models, this study systematically observed the expression profile changes of key PANoptosis genes in an animal model simulating a real pathological environment, providing solid experimental evidence for its clinical translational application.

This study has several limitations that must be acknowledged. Firstly, the relatively small sample size may affect the robustness and generalizability of the findings, necessitating validation in larger clinical cohorts to confirm the identified PANoptosis-related biomarkers in UC. Secondly, while the biomarker signature demonstrates high accuracy in distinguishing UC from healthy controls, its specificity against other intestinal inflammatory diseases, particularly Crohn’s disease, remains to be evaluated in dedicated differential diagnosis cohorts. This is a crucial next step for assessing its clinical utility. Additionally, the absence of wet lab experiments limits the ability to directly validate the functional roles of the identified hub genes in UC pathogenesis. Furthermore, the integration of multiple datasets carries inherent risks of batch effects, which may introduce technical variation. Future investigations would benefit from dedicated batch-effect adjustment strategies. This study provides new insights for the early diagnosis and personalized treatment of UC by systematically analyzing biomarkers related to PANoptosis, highlighting its innovation and practical significance. The six identified key biomarkers demonstrate potential clinical application prospects; future research should further focus on expanding sample sizes and **c**onducting multi-center validation, including specificity testing against relevant differential diagnoses, while integrating wet lab experiments (such as knockdown or overexpression of the identified biomarkers in cell or animal models) to delve deeper into the functions and mechanisms of these biomarkers, in order to offer more effective prevention and treatment strategies for UC patients.

Collectively, our research successfully identified six potential PANoptosis-related biomarkers for UC diagnosis through a comprehensive bioinformatics approach, including differential expression analysis, co-expression gene molecules analysis, and several machine learning techniques. The machine learning algorithms demonstrated strong diagnostic performance, with AUC values exceeding 0.800 in validation datasets. These results underscore the clinical promise of these biomarkers in enhancing diagnostic accuracy and understanding the underlying mechanisms of UC. Future studies should focus on larger clinical validations and functional assays to determine the pathophysiological signature of these biomarkers in UC.

## Data Availability

The original contributions presented in the study are included in the article/**Supplementary Material**. Further inquiries can be directed to the corresponding author/s.
