# Supplementary material for: A multi-machine learning framework identifies novel PANoptosis-related biomarkers and their immune landscape in ulcerative colitis: Insights from transcriptomics and experimental validation
Source: Front Immunol. 2026 Feb 18;17:1729942. doi: 10.3389/fimmu.2026.1729942 (PMC12956638; doi:10.3389/fimmu.2026.1729942)
Supplement: Supplementary file 1 [file SupplementaryFile1.zip › Suppl. Table 5.DOCX]

Table Supplementary 5. Diagnostic Performance Characteristics of the Six-Gene Significance Across Validation Datasets

| Dataset | Method | Accuracy | Balanced_accuracy | Sensitivity | Specificity | Precision | F1 |
| --- | --- | --- | --- | --- | --- | --- | --- |
| GSE47908 | NB | 0.833 | 0.756 | 0.600 | 0.911 | 0.692 | 0.643 |
| GSE47908 | SVM | 0.850 | 0.767 | 0.600 | 0.933 | 0.750 | 0.667 |
| GSE47908 | RF | 0.800 | 0.600 | 0.200 | 1.000 | 1.000 | 0.333 |
| GSE47908 | KNN | 0.933 | 0.889 | 0.800 | 0.978 | 0.923 | 0.857 |
| GSE47908 | LogitBoost | 0.833 | 0.733 | 0.533 | 0.933 | 0.727 | 0.615 |
| GSE38713 | NB | 0.800 | 0.841 | 1.000 | 0.682 | 0.650 | 0.788 |
| GSE38713 | SVM | 0.714 | 0.773 | 1.000 | 0.545 | 0.565 | 0.722 |
| GSE38713 | RF | 0.771 | 0.818 | 1.000 | 0.636 | 0.619 | 0.765 |
| GSE38713 | KNN | 0.800 | 0.841 | 1.000 | 0.682 | 0.650 | 0.788 |
| GSE38713 | LogitBoost | 0.771 | 0.818 | 1.000 | 0.636 | 0.619 | 0.765 |
