# Supplementary material for: A multi-machine learning framework identifies novel PANoptosis-related biomarkers and their immune landscape in ulcerative colitis: Insights from transcriptomics and experimental validation
Source: Front Immunol. 2026 Feb 18;17:1729942. doi: 10.3389/fimmu.2026.1729942 (PMC12956638; doi:10.3389/fimmu.2026.1729942)
Supplement: Supplementary file 1 [file SupplementaryFile1.zip › Suppl. Tables 1-4, Suppl. Figures.DOCX]

**Supplementary file**


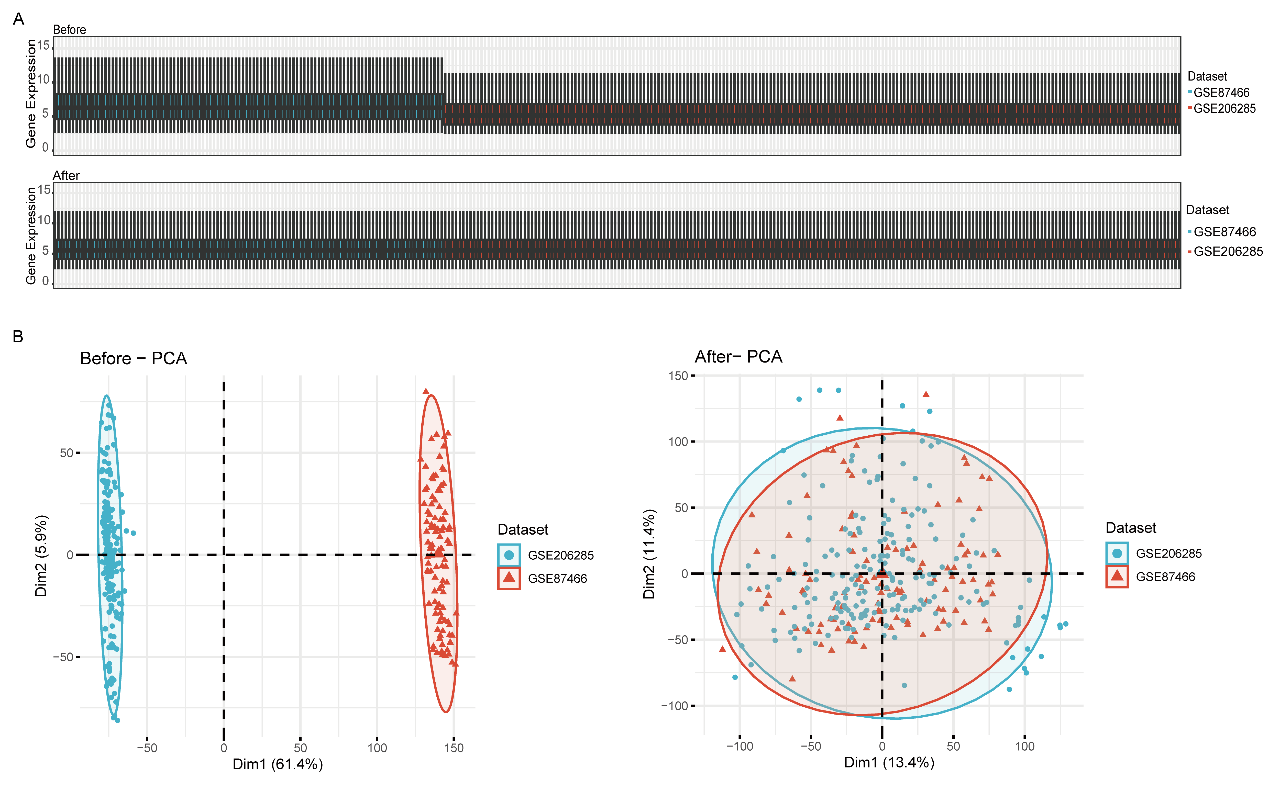


**Supplementary figure 1. Data preprocessing**. A. Boxplot before and after normalization of raw data between samples. B. PCA analysis before and after normalization of raw data between samples.

Table S1. KEGG top 10 pathways enrichment of 858 DEGs in UC

| **ID** | **Description** | **GeneRatio** | **BgRatio** | **p value** | **p.adjust** | **Count** |
| --- | --- | --- | --- | --- | --- | --- |
| hsa04061 | Viral protein interaction with cytokine and cytokine receptor | 24/455 | 100/8538 | 2.77E-10 | 6.83E-08 | 24 |
| hsa04060 | Cytokine-cytokine receptor interaction | 44/455 | 298/8538 | 4.70E-10 | 6.83E-08 | 44 |
| hsa04974 | Protein digestion and absorption | 23/455 | 105/8538 | 4.46E-09 | 4.32E-07 | 23 |
| hsa05150 | Staphylococcus aureus infection | 20/455 | 102/8538 | 3.20E-07 | 2.32E-05 | 20 |
| hsa04657 | IL-17 signaling pathway | 19/455 | 95/8538 | 4.57E-07 | 2.66E-05 | 19 |
| hsa04512 | ECM-receptor interaction | 18/455 | 89/8538 | 7.73E-07 | 3.75E-05 | 18 |
| hsa05323 | Rheumatoid arthritis | 18/455 | 95/8538 | 2.10E-06 | 8.75E-05 | 18 |
| hsa04151 | PI3K-Akt signaling pathway | 41/455 | 362/8538 | 3.22E-06 | 0.000117 | 41 |
| hsa04640 | Hematopoietic cell lineage | 18/455 | 100/8538 | 4.53E-06 | 0.000147 | 18 |
| hsa05146 | Amoebiasis | 18/455 | 103/8538 | 6.99E-06 | 0.000204 | 18 |

Table S2. Top10 GO enrichment of 858 DEGs

|  | ID | Description | GeneRatio | p.adjust | Count |
| --- | --- | --- | --- | --- | --- |
| BP | GO:0050900 | leukocyte migration | 70/767 | 3.97E-22 | 70 |
| BP | GO:0030595 | leukocyte chemotaxis | 51/767 | 2.09E-19 | 51 |
| BP | GO:0002237 | response to molecule of bacterial origin | 61/767 | 7.54E-18 | 61 |
| BP | GO:0006935 | chemotaxis | 69/767 | 1.00E-17 | 69 |
| BP | GO:0042330 | taxis | 69/767 | 1.02E-17 | 69 |
| BP | GO:0032496 | response to lipopolysaccharide | 58/767 | 2.43E-17 | 58 |
| BP | GO:0060326 | cell chemotaxis | 55/767 | 9.88E-17 | 55 |
| BP | GO:0007159 | leukocyte cell-cell adhesion | 62/767 | 4.32E-16 | 62 |
| BP | GO:0030593 | neutrophil chemotaxis | 31/767 | 9.66E-16 | 31 |
| BP | GO:0030198 | extracellular matrix organization | 53/767 | 1.00E-15 | 53 |
| CC | GO:0062023 | collagen-containing extracellular matrix | 81/787 | 4.95E-30 | 81 |
| CC | GO:0009897 | external side of plasma membrane | 60/787 | 1.45E-17 | 60 |
| CC | GO:0005604 | basement membrane | 25/787 | 7.72E-13 | 25 |
| CC | GO:0045177 | apical part of cell | 55/787 | 5.33E-11 | 55 |
| CC | GO:0005788 | endoplasmic reticulum lumen | 42/787 | 3.28E-10 | 42 |
| CC | GO:0030667 | secretory granule membrane | 40/787 | 4.53E-09 | 40 |
| CC | GO:0005581 | collagen trimer | 20/787 | 5.56E-09 | 20 |
| CC | GO:0016324 | apical plasma membrane | 46/787 | 5.56E-09 | 46 |
| CC | GO:0098644 | complex of collagen trimers | 10/787 | 9.04E-08 | 10 |
| CC | GO:0060205 | cytoplasmic vesicle lumen | 38/787 | 9.51E-08 | 38 |
| MF | GO:0005201 | extracellular matrix structural constituent | 42/767 | 7.86E-19 | 42 |
| MF | GO:0005539 | glycosaminoglycan binding | 38/767 | 4.26E-10 | 38 |
| MF | GO:0030020 | extracellular matrix structural constituent conferring tensile strength | 16/767 | 3.72E-09 | 16 |
| MF | GO:0008009 | chemokine activity | 16/767 | 1.24E-08 | 16 |
| MF | GO:0005178 | integrin binding | 27/767 | 3.15E-08 | 27 |
| MF | GO:0140375 | immune receptor activity | 26/767 | 4.66E-08 | 26 |
| MF | GO:0042379 | chemokine receptor binding | 18/767 | 1.03E-07 | 18 |
| MF | GO:0005125 | cytokine activity | 32/767 | 4.10E-07 | 32 |
| MF | GO:0019838 | growth factor binding | 23/767 | 6.84E-07 | 23 |
| MF | GO:0045236 | CXCR chemokine receptor binding | 9/767 | 9.45E-07 | 9 |

Table S3. GO enrichment of 53 PRGs-UC

|  | **ID** | **Description** | **GeneRatio** | **p.adjust** | **Count** |
| --- | --- | --- | --- | --- | --- |
| BP | GO:0032496 | response to lipopolysaccharide | 16/53 | 1.42E-12 | 16 |
| BP | GO:0050727 | regulation of inflammatory response | 17/53 | 1.42E-12 | 17 |
| BP | GO:0002237 | response to molecule of bacterial origin | 16/53 | 2.18E-12 | 16 |
| BP | GO:0007159 | leukocyte cell-cell adhesion | 16/53 | 1.18E-11 | 16 |
| BP | GO:0045765 | regulation of angiogenesis | 15/53 | 1.28E-11 | 15 |
| BP | GO:1901342 | regulation of vasculature development | 15/53 | 1.31E-11 | 15 |
| BP | GO:0006935 | chemotaxis | 16/53 | 3.49E-11 | 16 |
| BP | GO:0042330 | taxis | 16/53 | 3.49E-11 | 16 |
| BP | GO:0001819 | positive regulation of cytokine production | 16/53 | 7.77E-11 | 16 |
| BP | GO:0031349 | positive regulation of defense response | 15/53 | 6.45E-10 | 15 |
| CC | GO:0062023 | collagen-containing extracellular matrix | 12/53 | 1.22E-07 | 12 |
| CC | GO:0009897 | external side of plasma membrane | 10/53 | 3.76E-06 | 10 |
| CC | GO:0045121 | membrane raft | 8/53 | 2.52E-05 | 8 |
| CC | GO:0098857 | membrane microdomain | 8/53 | 2.52E-05 | 8 |
| CC | GO:0005925 | focal adhesion | 7/53 | 0.00272 | 7 |
| CC | GO:0030055 | cell-substrate junction | 7/53 | 0.00272 | 7 |
| CC | GO:0005788 | endoplasmic reticulum lumen | 6/53 | 0.002985 | 6 |
| CC | GO:0098636 | protein complex involved in cell adhesion | 3/53 | 0.00691 | 3 |
| CC | GO:0005769 | early endosome | 6/53 | 0.012033 | 6 |
| CC | GO:0034774 | secretory granule lumen | 5/53 | 0.016235 | 5 |
| MF | GO:0035325 | Toll-like receptor binding | 4/52 | 8.50E-06 | 4 |
| MF | GO:0005178 | integrin binding | 7/52 | 2.17E-05 | 7 |
| MF | GO:0005125 | cytokine activity | 8/52 | 2.17E-05 | 8 |
| MF | GO:0036041 | long-chain fatty acid binding | 3/52 | 0.000672 | 3 |
| MF | GO:0001221 | transcription coregulator binding | 5/52 | 0.000672 | 5 |
| MF | GO:0038187 | pattern recognition receptor activity | 3/52 | 0.004285 | 3 |
| MF | GO:0031625 | ubiquitin protein ligase binding | 6/52 | 0.006761 | 6 |
| MF | GO:0044389 | ubiquitin-like protein ligase binding | 6/52 | 0.006985 | 6 |
| MF | GO:0005504 | fatty acid binding | 3/52 | 0.006985 | 3 |
| MF | GO:0050786 | RAGE receptor binding | 2/52 | 0.006985 | 2 |

**Table S4. KEGG enrichment of 53 PRGs-UC**

| **ID** | **Description** | **GeneRatio** | **p.adjust** | **Count** |
| --- | --- | --- | --- | --- |
| hsa04064 | NF-kappa B signaling pathway | 9/47 | 6.81E-07 | 9 |
| hsa05417 | Lipid and atherosclerosis | 11/47 | 1.34E-06 | 11 |
| hsa04668 | TNF signaling pathway | 8/47 | 1.07E-05 | 8 |
| hsa05144 | Malaria | 6/47 | 1.07E-05 | 6 |
| hsa04657 | IL-17 signaling pathway | 7/47 | 2.39E-05 | 7 |
| hsa04061 | Viral protein interaction with cytokine and cytokine receptor | 7/47 | 2.83E-05 | 7 |
| hsa05205 | Proteoglycans in cancer | 9/47 | 3.05E-05 | 9 |
| hsa04620 | Toll-like receptor signaling pathway | 7/47 | 3.81E-05 | 7 |
| hsa04060 | Cytokine-cytokine receptor interaction | 10/47 | 6.83E-05 | 10 |
| hsa01521 | EGFR tyrosine kinase inhibitor resistance | 6/47 | 7.17E-05 | 6 |
| hsa05323 | Rheumatoid arthritis | 6/47 | 0.000177 | 6 |
| hsa05202 | Transcriptional misregulation in cancer | 7/47 | 0.001254 | 7 |
| hsa05169 | Epstein-Barr virus infection | 7/47 | 0.001396 | 7 |
| hsa04151 | PI3K-Akt signaling pathway | 9/47 | 0.001512 | 9 |
| hsa04672 | Intestinal immune network for IgA production | 4/47 | 0.001666 | 4 |
| hsa04014 | Ras signaling pathway | 7/47 | 0.002944 | 7 |
| hsa04370 | VEGF signaling pathway | 4/47 | 0.002989 | 4 |
| hsa05416 | Viral myocarditis | 4/47 | 0.005099 | 4 |
| hsa05167 | Kaposi sarcoma-associated herpesvirus infection | 6/47 | 0.005667 | 6 |
| hsa04510 | Focal adhesion | 6/47 | 0.006473 | 6 |
| hsa05133 | Pertussis | 4/47 | 0.006584 | 4 |
| hsa04015 | Rap1 signaling pathway | 6/47 | 0.006983 | 6 |
| hsa05418 | Fluid shear stress and atherosclerosis | 5/47 | 0.006983 | 5 |
| hsa05143 | African trypanosomiasis | 3/47 | 0.006983 | 3 |
| hsa04623 | Cytosolic DNA-sensing pathway | 4/47 | 0.006983 | 4 |
| hsa04010 | MAPK signaling pathway | 7/47 | 0.007217 | 7 |
| hsa05163 | Human cytomegalovirus infection | 6/47 | 0.008366 | 6 |
| hsa05332 | Graft-versus-host disease | 3/47 | 0.010916 | 3 |
| hsa04020 | Calcium signaling pathway | 6/47 | 0.01386 | 6 |
| hsa04625 | C-type lectin receptor signaling pathway | 4/47 | 0.01386 | 4 |
| hsa05145 | Toxoplasmosis | 4/47 | 0.016946 | 4 |
| hsa05134 | Legionellosis | 3/47 | 0.017887 | 3 |
| hsa04670 | Leukocyte transendothelial migration | 4/47 | 0.01806 | 4 |
| hsa04062 | Chemokine signaling pathway | 5/47 | 0.018964 | 5 |
| hsa05321 | Inflammatory bowel disease | 3/47 | 0.025981 | 3 |
| hsa05211 | Renal cell carcinoma | 3/47 | 0.029757 | 3 |
| hsa05230 | Central carbon metabolism in cancer | 3/47 | 0.030115 | 3 |
| hsa03320 | PPAR signaling pathway | 3/47 | 0.035382 | 3 |
| hsa04810 | Regulation of actin cytoskeleton | 5/47 | 0.035702 | 5 |
| hsa05140 | Leishmaniasis | 3/47 | 0.03738 | 3 |
| hsa05171 | Coronavirus disease - COVID-19 | 5/47 | 0.037694 | 5 |
| hsa04148 | Efferocytosis | 4/47 | 0.040032 | 4 |
| hsa04932 | Non-alcoholic fatty liver disease | 4/47 | 0.040032 | 4 |
| hsa01523 | Antifolate resistance | 2/47 | 0.042587 | 2 |
| hsa05132 | Salmonella infection | 5/47 | 0.042587 | 5 |
| hsa05235 | PD-L1 expression and PD-1 checkpoint pathway in cancer | 3/47 | 0.046302 | 3 |
| hsa04662 | B cell receptor signaling pathway | 3/47 | 0.046684 | 3 |
| hsa05164 | Influenza A | 4/47 | 0.049787 | 4 |
